# Social network distribution of HIV self-tests among MSM in Australia: a prospective, non-randomised trial

**DOI:** 10.1016/j.lanwpc.2026.101839

**Published:** 2026-03-24

**Authors:** Ying Zhang, Cheryl C. Johnson, Michael Traeger, Judith A. Dean, Eric P.F. Chow, Weiming Tang, Mark Stoové, Jason J. Ong

**Affiliations:** a School of Translational Medicine, Faculty of Medicine, Nursing and Health Sciences, Monash University, Melbourne, Victoria, Australia; b Melbourne Sexual Health Centre, Bayside Health, Melbourne, Victoria, Australia; c Global HIV, Hepatitis and STI Programmes, World Health Organization, Geneva, Switzerland; d Burnet Institute, Melbourne, Australia; e School of Public Health and Preventive Medicine, Monash University, Melbourne, Australia; f School of Public Health, Faculty of Health, Medicine and Behavioural Sciences, The University of Queensland, Brisbane, Queensland, Australia; g Centre for Epidemiology and Biostatistics, Melbourne School of Population and Global Health, The University of Melbourne, Melbourne, Victoria, Australia; h Department of Medicine, University of North Carolina at Chapel Hill, Chapel Hill, NC, USA; i School of Public Health, University of North Carolina at Chapel Hill, Chapel Hill, NC, USA; j Australian Research Centre in Sex, Health and Society, La Trobe University, Melbourne, Australia; k Faculty of Infectious and Tropical Diseases, London School of Hygiene and Tropical Medicine, London, United Kingdom

**Keywords:** HIV, Self-test, Men who have sex with men, Social network, Peer

## Abstract

**Background:**

HIV testing uptake among men who have sex with men (MSM) in Australia remains suboptimal, with lapses and extended testing intervals persisting despite established guidelines. Novel HIV testing strategies address these gaps, particularly among MSM underserved by clinic-based services. We evaluated uptake of HIV self-testing (HIVST) distributed using social network-based strategies among MSM in Australia.

**Methods:**

In this prospective, non-randomised trial (ACTRN12625000342415), HIV-undiagnosed MSM aged ≥18 years were recruited online on a rolling basis between Jan 30 and April 28, 2025. Participants comprised both test promoters (initially recruited individuals who received HIVST kits for personal use and distribution) and recipients (individuals who received a kit from a promoter). Test promoters each received four HIVST kits—one for personal use and three for distribution. Participants were invited to complete an online survey within three months of kit receipt. Recipients who completed the survey could enrol as second-wave test promoters and receive additional kits. Primary outcome: HIVST use within 7 days of receipt; secondary outcomes: use within 24 h, feasibility and acceptability. Associations with use were estimated using Poisson regression.

**Findings:**

Ninety-one test promoters received 364 HIVST kits (91 for personal use, 273 for distribution). All promoters used their own kit and completed the follow-up survey (100%). Among the first-wave recipients, 245 kits were survey-verified as used. Eight recipients subsequently enrolled as second-wave test promoters, distributing additional kits and yielding 15 survey-verified secondary recipients. In total, 351 participants completed follow-up surveys. Of respondents, 280 (80%) used the HIVST within 7 days, including 181 (52%) within 24 h. Use within 7 days was more common among participants without government-subsidised healthcare (adjusted rate ratio 1.16, 1.05–1.28; p = 0.004), those not tested in the past 3 months (3–6 months, 1.22 [1.02–1.45], p = 0.026; 7–12 months, 1.36 [1.16–1.60], p < 0.001; >12 months, 1.30 [1.11–1.53], p = 0.001; never tested, 1.31 [1.11–1.55], p = 0.002), and those spending little (1.36 [1.10–1.68]; p = 0.004) or no time (1.33 [1.06–1.66]; p = 0.015) with LGBTQ+ peers. Most participants found HIVST easy to use (99%) and would reuse it (88%).

**Interpretation:**

Social network-based distribution of HIVST is feasible and acceptable, with high HIVST kit usability and rapid uptake among MSM, supporting equitable HIV testing access.

**Funding:**

Australian National Health and Medical Research Council (NHMRC) Emerging Leadership Investigator Grant and an Australian Government Research Training Program scholarship.

**Research in context**

**Evidence before this study**

We searched PubMed for articles published January 1, 2010–October 31, 2025, using the terms “HIV self-testing”, “social network distribution”, “men who have sex with men”, and “Australia”. Australian studies were reviewed to identify evidence specific to local HIV testing strategies and implementation contexts. This search identified limited Australian evidence on social network-based HIV self-testing (HIVST), with most initiatives relying on online ordering or clinic-mediated distribution. To contextualise this local evidence gap, we also reviewed international studies. Evidence from China and sub-Saharan Africa shows that social- and peer-network distribution approaches can increase HIVST uptake and extent reach to underserved communities by improving kit availability and access within trusted social networks. Several studies report that delivery by trusted peers enhances social credibility and provides practical support, facilitating uptake, whereas some have noted that receiving kits from friends or partners may cause discomfort or perceptions of mistrust, reflecting the complex social dynamics of peer-led distribution. Despite this growing international literature, evaluation of social network-based HIVST strategies in high-income settings such as Australia remains limited, and mobile populations, including overseas-born and migrant MSM, who remain under-represented in clinic-based testing. To our knowledge, social network-based HIVST distribution has not previously been assessed at national scale in a high-income context with this degree of demographic and social heterogeneity.

**Added value of this study**

To our knowledge, this is the first demonstration project in Australia evaluating social network-based distribution of HIVST kits among MSM. Although participation was open to all eligible MSM, the study demonstrates the potential of this approach to reach subgroups that face barriers to clinic-based testing, including overseas-born and Medicare-ineligible MSM, individuals who do not speak English as a first language, and those with limited connection to LGBTQI+ social networks. We assessed not only overall usage but also the time from kit receipt to self-testing, demonstrating that a majority of MSM used the self-test within 24 h of receiving it (and most within a week)—an indicator of responsiveness that has been rarely reported in previous HIVST studies. Despite operating in a context of universal healthcare and high baseline testing coverage, this social network-driven approach achieved high acceptability and engaged a broad spectrum of MSM in Australia. Together, these findings extend international evidence from low- and middle-income settings to a high-income, multicultural context and illustrate how social network strategies can complement existing testing services and support more equitable access to HIV testing.

**Implications of all the available evidence**

In this Australian demonstration study, social network-based distribution of HIVST achieved rapid and acceptable uptake predominantly among MSM already engaged with HIV prevention and testing services. In settings characterised by high baseline testing coverage and widespread PrEP use, the primary contribution of this approach appears to be improved convenience, timeliness, and flexibility of testing rather than identification of previously undiagnosed HIV infection; it should therefore be considered a complementary strategy alongside clinic- and online-based testing models. Although most participants had prior testing experience, social network-based HIVST may retain value for reaching under-tested or never-tested MSM when programmes are intentionally designed to do so, as social networks can facilitate access through trust, peer endorsement, and informal pathways, particularly for individuals with limited engagement in conventional services. Future implementations should prioritise targeted recruitment and culturally appropriate support to better evaluate the equity and case-finding potential of this approach in high-income settings.

## Introduction

HIV testing is fundamental to achieving the UNAIDS 95-95-95 targets, providing the gateway to both prevention and treatment. Despite major progress in global testing initiatives, undiagnosed HIV infection remains a key barrier to elimination, sustaining avoidable transmission and delaying access to care. Early diagnosis and prompt initiation of antiretroviral therapy (ART) are therefore critical, highlighting the need for strategies that increase testing frequency and accessibility among those at greatest risk.

In Australia, national guidelines recommend testing every three months for sexually active men who have sex with men (MSM).^1^ However, testing uptake remains suboptimal. Among MSM not using pre-exposure prophylaxis (PrEP), only around half report annual HIV testing,^2^ and nearly half of new HIV diagnoses among MSM are classified as late.^3^ Testing disparities are particularly pronounced among overseas-born MSM, including those from Southeast Asia and Latin America, who experience higher levels of undiagnosed infection and delayed engagement with care.^3^ Contributing factors include lack of Medicare eligibility, limited familiarity with health services, language and cultural barriers, confidentiality concerns within small migrant communities, and weaker connection to LGBTQ+ social networks.^4^^,^^5^ These persistent barriers highlight the limitations of clinic-based testing models, even within a universal healthcare system.

HIV self-testing (HIVST) offers a complementary approach that can expand testing coverage among populations least reached by conventional services. Following WHO recommendations in 2016, more than 100 countries have integrated HIVST into national HIV testing programmes.^6^ In Australia, HIVST was approved by the Therapeutic Goods Administration in 2018. Evidence across diverse settings shows that HIVST can increase testing frequency, accelerates time to diagnosis, and supports linkage-to-care, particularly when distributed through peer-led or social network-based models.^7^, ^8^, ^9^ However, most Australian HIVST initiatives to date have relied on online ordering or clinic-mediated distribution, with limited evaluation of alternative delivery models that leverage social networks.

Social network-based HIVST distribution refers to the provision of self-test kits through existing social or sexual ties, whereby individuals (“test promoters”) distribute kits to people within their personal networks, such as friends, partners, or acquaintances. By leveraging interpersonal trust and social proximity, this approach may enhance acceptability and reach individuals less engaged with formal health services. International evidence, predominately from low- and middle-income settings, suggests that network-based approaches can increase HIV testing uptake and extend reach to first-time and infrequent testers.^4^^,^^5^ WHO guidance and systematic reviews further support the feasibility and effectiveness of network-based approaches in identifying undiagnosed infections and potentially improving equity in testing access.^8^^,^^10^, ^11^, ^12^, ^13^, ^14^, ^15^

Evidence from high-income settings remains more limited but is emerging. Studies from the United States have demonstrated the feasibility of peer- and network-based HIVST distribution among MSM, including programmes that leveraged social and sexual networks of peer recruiters and PrEP users to engage network members in HIV testing.^16^^,^^17^ These studies provide relevant points of comparison for Australia, given similarities in health systems, HIV epidemiology, and prevention coverage, but also highlight the need for context-specific evaluation of network diffusion and implementation.

Building on this evidence gap, we conducted a nationally recruited demonstration study to evaluate a social network-based HIVST distribution model among MSM in Australia. The study aimed to assess (1) HIVST uptake and timeliness of use, (2) feasibility and acceptability of the model, and (3) predictors of strong test promoter performance, defined as the proportion of distributed HIVST kits with a corresponding recipient post-test survey completed.

## Methods

### Study design

**Fig. 1:**
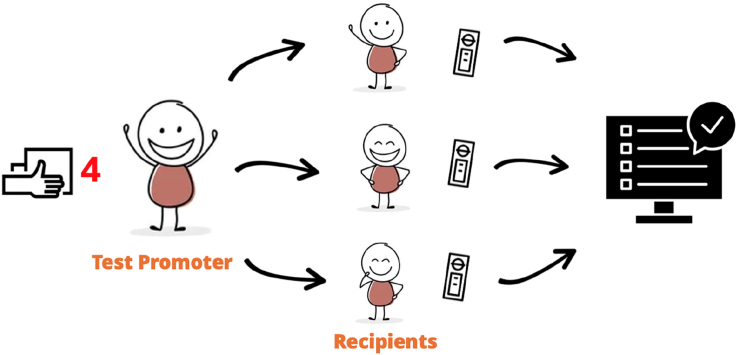
Trial process.

This is a prospective, non-randomised demonstration study evaluating HIVST kits delivered through a social network approach. The intervention recruited initial participants (“test promoters”), each provided with four HIVST kits—one for personal use and three to distribute to social or sexual contacts (“recipients”). All participants were provided finger-prick HIV self-tests (Atomo HIV Self-Test, Mylan-Atomo Diagnostics) and asked to complete online post-test survey within three months of kit receipt (Fig. 1). Recipients could subsequently enrol as test promoters, generating a second recruitment wave (wave 2) that followed identical procedures; no further waves were initiated.

### Recruitment

Participants were recruited nationally between January 30 and April 28, 2025, through community organisations (e.g., Australia and New Zealand Tongzhi Rainbow Alliance, Thorne Harbour Health), universities, social media, and sexual health clinics (e.g., Melbourne Sexual Health Centre). Interested individuals accessed an eligibility survey (Qualtrics) via QR code and received study information and consent forms by email. Follow-up continued until July 31, 2025.

### Study population

Eligible participants were MSM, aged ≥18 years, living in Australia, and without a known HIV diagnosis (unknown status permitted). Country of birth was not restricted. Two groups were enrolled: test promoters and their recipients; primary analyses classified participants according to their initial role.

### Sample size

This study was designed as a national demonstration project rather than a hypothesis-testing trial. The sample size was therefore determined to provide reasonable precision around the primary outcome: self-reported HIVST use within 7 days of kit receipt among participants with a completed post-test survey. Assuming a timely-use proportion of approximately 75–80%, a sample of around 300 survey-verified participants was expected to yield a two-sided 95% confidence interval with a margin of error of approximately ±4–5%.

Verification of HIVST use relied on completion of follow-up post-test surveys linked to unique kit identifiers. Nonresponse and incomplete verification were therefore considered in interpretation rather than formally inflated in the sample size calculation. Given the demonstration nature of the study and the small size of most social networks (maximum three recipients per test promoter), clustering was not explicitly incorporated into the sample size rationale. Study reporting followed CONSORT extension guidance^18^ and the TREND checklist.^19^

### Procedures

Test promoters provided written consent and were mailed four HIVST kits. Each package included a HIVST kit with the manufacturer's components, a culturally tailored study information booklet, instructions for use and interpretation (including guidance for reactive and invalid results), a condom, a lollipop, and an adhesive bandage. Packaging was plain and unmarked to protect confidentiality.

Study materials were developed through a community crowdsourcing contest to ensure cultural appropriateness. Participants received instructions via email and in the booklet, directing them to a study webpage containing an instructional video, FAQs, counselling resources, and the post-test survey (accessible via URL or QR code on each kit). Test promoters were instructed to use one kit themselves and distribute the remaining three to adults MSM (aged ≥18 years) residing in Australia, with an emphasis on reaching overseas-born MSM.

Each kit carried a unique identifier (“ST number”) linking de-identified survey responses to the distributing test promoter and recruitment wave. Promoters collected no personal information about recipients. Recipients accessed the study webpage independently and provided electronic consent before completing the post-test survey.

HIVST distribution and use were assessed through a kit-level cascade. Kits were classified as: (1) issued (mailed to test promoters), (2) delivered (confirmed by postal tracking), and (3) survey-verified, defined as completion of a post-test survey using the kit's unique identifier. Completion of the post-test survey required participants to report their HIVST result and therefore implied that the HIVST had been used. For conservative estimates, kits without a matched survey were treated as non-use.

To support timely use and distribution of HIVST kits, test promoters received automated reminder emails after kit delivery and at approximately 1 week, 2.5 weeks, 1 month, 2 months, and 3 months following receipt of the kits. These reminders encouraged promoters to use their own HIVST kit, distribute remaining kits to eligible peers, and prompt recipients to use the HIVST.

To minimise duplicate participation and fraudulent submissions, access to the post-test survey was restricted to individuals in possession of a valid, unused kit identifier, and only one submission was permitted per identifier. Survey metadata, including timestamps and IP addresses, were routinely monitored to identify potential duplicate or suspicious entries; where duplicate submissions were detected, these were consolidated and counted as a single submission in the analytic dataset.

Participants were asked to complete post-test surveys within three months of kit receipt. Surveys captured demographics, HIV testing history, sexual behaviours, community connectedness, HIVST result, and feasibility and acceptability measures (including ease of use and willingness to reuse HIVST).

Test promoters received AUD20 for completing their own post-test survey, AUD20 per completed recipient survey (up to three), and an additional AUD20 bonus if all three recipients completed surveys. Reimbursement was therefore based on survey return rather than self-reported distribution. Distribution verification relied exclusively on matched recipient survey submissions. Recipients enrolling as test promoters received four additional kits and were analysed as part of the second wave. Repeat self-testing was not required.

### Outcomes

The primary outcome was self-reported HIVST use within seven days of kit receipt, selected to capture prompt engagement as an indicator of rapid uptake.

Secondary outcomes were HIVST use within 24 h, proportion of reactive results, feasibility and acceptability measures, and network reach, defined as the proportion of kits reaching second-wave recipients.

Time to HIVST use was assessed using a self-reported categorical measure in the post-test survey, which asked participants how long after receiving the HIVST kit they performed the test (e.g., within 24 h, within 7 days, or later). Because exact receipt dates were not collected, time-to-use outcomes were derived from these categories rather than calendar intervals. Surveys with missing responses to the time-to-use question were excluded from analyses of timeliness.

### Data analysis

Two incomplete surveys containing only age data were excluded. Participants were classified by index role for analyses of initial HIVST use; for network reach and promoter performance, recipients who became test promoters were analysed as promoters.

Associations between HIVST use within 7 days and 24 h and prespecified covariates were assessed using Poisson regression with a log link and robust variance estimation, reported as adjusted risk ratios (aRRs). Poisson regression was selected because HIVST uptake was common and logistic regression would overestimate effect sizes.^20^ Prespecified covariates included age, education, first language, Medicare eligibility, state of residence, time since last HIV test, and LGBTQ+ community connectedness. Medicare eligibility was used as a proxy for healthcare access given its role in providing subsidised medical services to Australian citizens, permanent residents, and select visa holders. All prespecified covariates were entered into multivariable models, with block-wise backward elimination (p > 0.20) used to retain variables associated with the outcome or acting as confounders. Missing data were minimal (<4% for all covariates) and were handled using complete-case analysis.

Clustering by network was not formally modelled. This decision was based on the network structure, as most promoters distributed a small number of kits (maximum three), networks were small with minimal overlap, and outcomes were measured at the individual level. Under these conditions, the impact of intra-network correlation on variance estimates was considered unlikely to materially affect inference. Multivariable models included all prespecified covariates, with block-wise backward elimination (p > 0.20) used to retain variables with evidence of association or confounding.

Feasibility was defined as the extent to which the HIVST distribution model could be delivered and used as intended in real-world conditions. Feasibility was assessed across three domains: (1) usability, captured through ease-of-use ratings and survey completion following kit use; (2) acceptability, measured through willingness to reuse HIVST; and (3) compatibility, reflected in willingness to use HIVST between clinic visits. Indicators were summarised descriptively using 5-point Likert scales. Ease-of-use responses ranged from “extremely easy” to “not at all easy.” For regression analyses, Likert responses were collapsed into high, moderate, and low categories to address sparse cells and improve interpretability, and analysed using ordinal logistic regression. Proportional-odds assumptions were assessed graphically and using score tests; where assumptions were not met, alternative model specifications were explored, with consistent results.

Sensitivity analyses using Poisson regression examined predictors of strong test promoter performance, defined as the proportion of HIVST kits with a corresponding recipient survey completed (maximum three per promoter). Because reimbursement was tied to recipient survey completion, the number of completed surveys was used as a proxy for kit distribution.

Descriptive analyses summarised demographic, behavioural, and testing characteristics of test promoters and recipients across recruitment waves. Categorical variables were compared using χ^2^ or Fisher's exact tests, and continuous variables using the Wilcoxon rank-sum test. All analyses were conducted using STATA (version 19, StataCorp LLC, United States), and statistical significance was assessed at a two-sided α level of 0.05.

The registered study protocol included both quantitative and qualitative components. While the quantitative outcomes reported here align with the primary and secondary endpoints of HIVST uptake, timeliness, feasibility, and acceptability, the qualitative interviews exploring participant experiences and perceptions are reported separately in a standalone manuscript. This paper therefore focuses exclusively on the quantitative evaluation of the social network-based HIVST distribution model.

### Ethics approval

The study received ethical approval from the Alfred Hospital Human Research Ethics Committee, Melbourne, Australia (Project 322/24), and was registered with the Australia New Zealand Clinical Trials Registry (ACTRN12625000342415). The studies were conducted in accordance with the local legislation and institutional requirements. The participants provided their written informed consent to participate in this study.

### Role of funding source

The funders of the study had no role in study design, data collection, data analysis, data interpretation, or writing of the report.

## Results

In the initial wave, we recruited 91 test promoters and issued 364 HIVST kits in total, comprising one kit for personal use per promoter (n = 91) and 273 kits intended for distribution to recipients. Kit use was verified through completion of a post-test survey; survey completion required reporting an HIVST result and therefore implied test use. All test promoters used their own kit and completed the survey (100% follow-up). Among first-wave recipients, 245 of the 273 kits intended for distribution were survey-verified as used, corresponding to a recipient survey verification proportion of 90%. Overall, 336 of 364 kits issued in the first wave were survey-verified (92%).

Eight first-wave recipients subsequently enrolled as second-wave test promoters. These second-wave promoters received 24 additional HIVST kits for distribution, but were not required to repeat HIVST or complete an additional post-test survey themselves. Distribution by second-wave promoters yielded 15 survey-verified secondary recipients (63%), indicating onward diffusion within social networks. Because completion of the post-test survey required reporting an HIVST result, there were no survey-verified kits that were not used.

Across both waves, a total of 388 HIVST kits were issued (364 in the first wave and 24 in the second wave), and 351 post-test surveys were completed, yielding an overall survey verification proportion of 90.4%. Of all issued kits, 280 (72%) were reported used within 7 days of receipt. Under a conservative assumption treating all kits without a matched post-test survey as non-use, the minimum estimated proportion of kits used within 7 days was 72% (280/388).

**Fig. 2:**
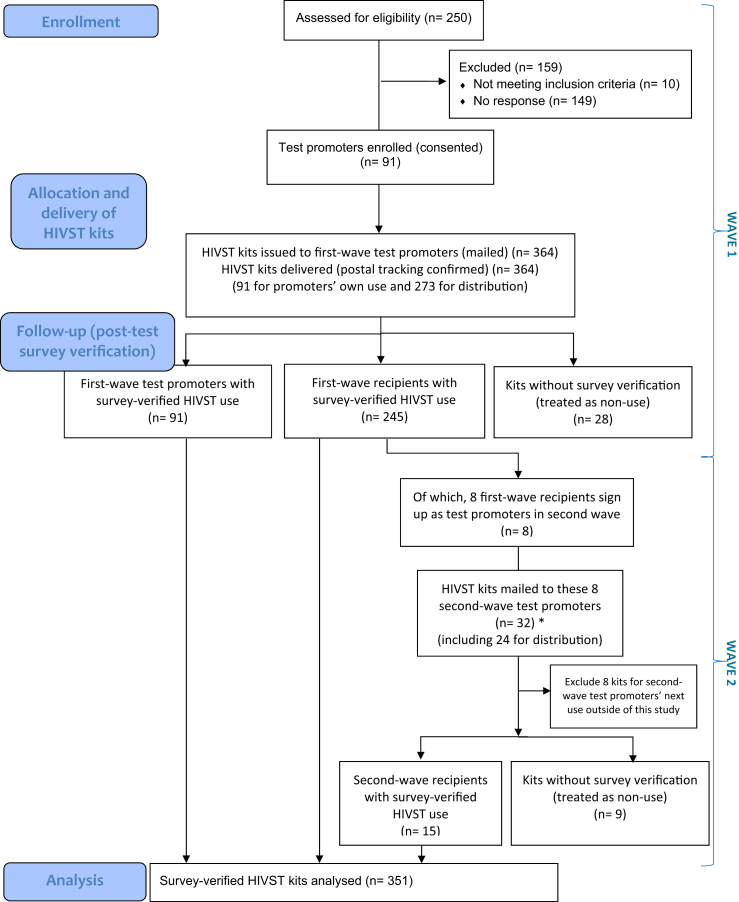
Participant flow and kit-level cascade of HIV self-testing distribution. ∗ Recipients who enrolled as test promoters received one HIVST kit for future personal use outside the trial. NB: Completion of the post-test survey required reporting of an HIVST result and therefore implied HIVST use. Kits without a matched survey were conservatively treated as non-use.

Among survey-verified recipient kits, 260 were used by recipients, comprising 245 first-wave recipients and 15 s-wave recipients. In total, 351 valid post-test surveys were analysed, including 91 test promoters and 260 recipients (Fig. 2).

**Fig. 3:**
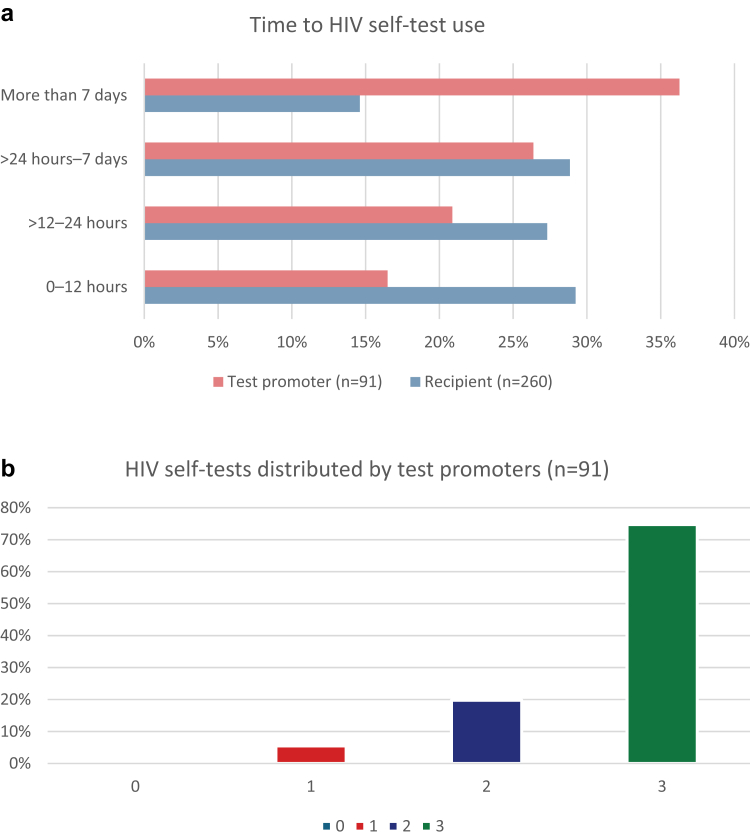
Time to HIV self-test use and kit distribution by participant role. (a) Time to first HIV self-test use after kit availability, by participant role. The distribution of time to first use is shown across mutually exclusive, non-overlapping intervals (0–12 h, >12–24 h, >24 h–7 days, and >7 days), comparing test promoters (n = 91) and recipients (n = 260). Percentages are calculated within each role, such that each role's categories sum to 100%. (b) Distribution of HIVST kits among test promoters, showing the proportion of promoters by number of kits distributed.

The mean age of test promoters was 35.3 years (SD: 9.56), compared with 32.1 years (SD: 8.08) among recipients. Baseline characteristics are shown in Table 1 and time-to-use distributions by participant role are presented in Fig. 3a. Participants were predominantly recruited from Victoria and New South Wales, with limited representation from other Australian states and territories (Table 1). Nearly all participants (97%) reported a prior HIV test, and over half (58%) reported current use of PrEP.

**Table 1:** Participant demographics by role at baseline index (N = 351).

	Total (N = 351)	
	Test promoter (N = 91)	Recipient (N = 260)
Age (years)	n (%)	n (%)
18–24	7 (7.7%)	46 (17.7%)
25–34	38 (41.8%)	131 (50.4%)
≥35	46 (50.5%)	83 (31.9%)
Country of birth		
Australia	24 (26.4%)	35 (13.5%)
Overseas	67 (73.6%)	225 (86.5%)
State		
Australian Capital Territory	1 (1.1%)	2 (0.8%)
New South Wales	11 (12.1%)	38 (14.6%)
Queensland	3 (3.3%)	9 (3.5%)
South Australia	1 (1.1%)	3 (1.2%)
Victoria	74 (81.3%)	203 (78.1%)
Western Australia	1 (1.1%)	5 (1.9%)
Medicare status^a^		
Medicare-eligible	64 (70.3%)	139 (53.5%)
Medicare-ineligible	27 (29.7%)	121 (46.5%)
First language other than English		
No	26 (28.6%)	57 (21.9%)
Yes	65 (71.4%)	203 (78.1%)
HIV testing recency		
<3 months ago	51 (56.0%)	63 (24.2%)
3–6 months ago	16 (17.6%)	71 (27.3%)
7–12 months ago	15 (16.5%)	61 (23.5%)
Over 12 months ago	9 (9.9%)	59 (22.7%)
Never tested for HIV	0 (0%)	7 (2.7%)
Education level		
High school	3 (3.3%)	23 (8.8%)
Certificate/Diploma	12 (13.2%)	24 (9.2%)
Bachelor	38 (41.8%)	134 (51.5%)
Postgraduate	38 (41.8%)	78 (30.0%)
Community connectedness (*How much of your social time do you spend with LGBTQ+ friends or community?*)		
Almost all of the time	5 (5.5%)	34 (13.1%)
Most of the time	36 (39.6%)	67 (25.8%)
Some of the time	39 (42.9%)	101 (38.8%)
A little of the time	11 (12.1%)	52 (20.0%)
None of the time	0 (0%)	6 (2.3%)
PrEP use		
Daily PrEP	32 (35.2%)	56 (21.5%)
On demand PrEP	39 (42.9%)	95 (36.5%)
Lapsed^b^	4 (4.4%)	29 (11.2%)
Never taken PrEP	16 (17.6%)	80 (30.8%)
Number of sexual partners in last 12 months		
0–1	5 (5.5%)	46 (17.7%)
2–5	26 (28.6%)	85 (32.7%)
6–10	19 (20.9%)	60 (23.1%)
>10	41 (45.1%)	69 (26.5%)

HIV, human immunodeficiency virus; LGBTQ+, lesbian, gay, bisexual, transgender and queer people; PrEP, pre-exposure prophylaxis.

a Australia's Medicare is a publicly funded universal healthcare system that provides free or subsidised access to medical services, hospital care, and prescription medications for Australian citizens and permanent residents.

b Participants self-identifying as having previously taken PrEP but not currently using it were categorised as “lapsed”.

Of the 351 participants, 280 (80%) reported using the HIVST within 7 days of receipt and 181 (52%) within 24 h (Table S1). In multivariable Poisson regression (Table 2), Medicare-ineligible participants were more likely to use the kit within 7 days than those with Medicare coverage (aRR 1.16, 1.05–1.28; p = 0.004). Time since last HIV test demonstrated a clear gradient: compared with participants who had tested within the previous 3 months, those with longer intervals were more likely to test promptly—3–6 months (1.22, 1.02–1.45; p = 0.026), 7–12 months (1.36, 1.16–1.60; p < 0.001), >12 months (1.30, 1.11–1.53; p = 0.001), and never tested (1.31, 1.11–1.55; p = 0.002). Lower community connectedness was also associated with timely use: participants spending little (1.36, 1.10–1.68; p = 0.004) or no time (1.33, 1.06–1.66; p = 0.015) with LGBTQ+ peers were more likely to use the kit within 7 days than those spending most or all of their time with LGBTQ+ peers.

**Table 2:** Multivariable Poisson regression of factors associated with HIVST use within 7 days (N = 351).

	HIVST use within 7 days, n/N (%)	Univariate		Multivariable	
		Risk ratio	p-value	Adjusted risk ratio	p-value
Role					
Test promoter	58/91 (63.7)	Ref	Ref	Ref	Ref
Recipient	222/260 (85.4)	1.34 (1.14–1.58)	<0.001	1.19 (1.02–1.39)	0.027
Age (years)					
18–24	49/53 (92.5)	Ref	Ref	Ref	Ref
25–34	141/169 (83.4)	0.89 (0.81–0.99)	0.037	1.03 (0.95–1.16)	0.364
≥35	90/129 (69.8)	0.75 (0.66–0.86)	<0.001	0.96 (0.82–1.11)	0.564
State					
Australian Capital Territory	3/3 (100.0)	Ref	Ref	NA	NA
New South Wales	38/49 (77.6)	0.78 (0.67–0.90)	0.001	NA	NA
Queensland	12/12 (100.0)	NA	NA	NA	NA
South Australia	3/4 (75.0)	0.75 (0.42–1.32)	0.320	NA	NA
Victoria	218/277 (78.7)	0.79 (0.74–0.83)	<0.001	NA	NA
Western Australia	6/6 (100.0)	NA	NA	NA	NA
Medicare status^a^					
Medicare-eligible	145/203 (71.4)	Ref	Ref	Ref	Ref
Medicare-ineligible	135/148 (91.2)	1.28 (1.15–1.41)	<0.001	1.16 (1.05–1.28)	0.004
First language other than English					
No	67/83 (80.7)	Ref	Ref	NA	NA
Yes	213/268 (79.5)	0.98 (0.87–1.11)	0.745	NA	NA
HIV testing recency					
<3 months ago	69/113 (61.1)	Ref	Ref	Ref	Ref
3–6 months ago	71/87 (81.6)	1.33 (1.18–1.59)	0.001	1.22 (1.02–1.45)	0.026
7–12 months ago	70/76 (92.1)	1.50 (1.28–1.77)	<0.001	1.36 (1.16–1.60)	<0.001
Over 12 months ago	63/68 (92.6)	1.64 (1.41–1.89)	<0.001	1.30 (1.11–1.53)	0.001
Never tested for HIV	7/7 (100.0)	1.52 (1.29–1.78)	<0.001	1.31 (1.11–1.55)	0.002
Education level attained					
Bachelor	147/172 (85.5)	Ref	Ref	NA	NA
High school	23/26 (88.5)	1.04 (0.89–1.21)	0.657	NA	NA
Certificate/Diploma	27/36 (75.0)	0.88 (0.72–1.07)	0.198	NA	NA
Postgraduate	82/116 (70.7)	0.83 (0.72–0.94)	0.005	NA	NA
Community connectedness (*How much of your social time do you spend with LGBTQ+ friends or community?*)					
Almost all of the time	26/39 (66.7)	Ref	Ref	Ref	Ref
Most of the time	72/103 (69.9)	1.05 (0.82–1.35)	0.717	1.17 (0.92–1.49)	0.192
Some of the time	116/140 (82.9)	1.24 (0.98–1.57)	0.069	1.24 (1.00–1.53)	0.050
A little of the time	60/63 (95.2)	1.43 (1.14–1.80)	0.002	1.36 (1.10–1.68)	0.004
None of the time	6/6 (100.0)	1.50 (1.20–1.87)	<0.001	1.33 (1.06–1.66)	0.015
PrEP use					
Daily PrEP	64/88 (72.7)	Ref	Ref	NA	NA
On demand PrEP	102/134 (76.1)	1.05 (0.89–1.23)	0.575	NA	NA
Lapsed^b^	29/33 (87.9)	1.20 (1.01–1.45)	0.040	NA	NA
Never taken PrEP	85/96 (88.5)	1.20 (1.03–1.40)	0.015	NA	NA
Number of sexual partners in last 12 months					
0–1	43/51 (84.3)	Ref	Ref	NA	NA
2–5	94/111 (84.7)	0.99 (0.86–1.15)	0.932	NA	NA
6–10	61/79 (77.2)	0.92 (0.77–1.08)	0.307	NA	NA
>10	82/110 (74.5)	0.88 (0.75–1.04)	0.135	NA	NA

Adjusted risk ratios (aRRs) estimated using multivariable Poisson regression. Outcome coded as 1 = HIV self-test used within 7 days of kit receipt, 0 = >7 days.

HIV, human immunodeficiency virus; LGBTQ+, lesbian, gay, bisexual, transgender and queer people; NA, not applicable; PrEP, pre-exposure prophylaxis; Ref, reference level.

a Australia's Medicare is a publicly funded universal healthcare system that provides free or subsidised access to medical services, hospital care, and prescription medications for Australian citizens and permanent residents.

b Participants self-identifying as having previously taken PrEP but not currently using it were categorised as “lapsed”.

Predictors of testing within 24 h showed similar patterns. Medicare ineligibility (1.36, 1.11–1.67; p = 0.004) and longer time since last HIV test were associated with immediate use—3–6 months (1.62, 1.13–2.31; p = 0.008), 7–12 months (2.19, 1.57–3.40; p < 0.001), >12 months (2.02, 1.41–2.99; p < 0.001), and never tested (2.59, 1.79–3.74; p < 0.001). Participants with a non-English first language were less likely to use the kit within 24 h (0.72, 0.59–0.89; p = 0.001; Table S2). Analyses restricted to recipients yielded similar findings (Tables S3 and S4).

Perceived feasibility and acceptability were high (Figures S1–S3). Nearly all participants (348/351, 99%) reported that HIVST was easy to use: 117 (33%) rated it extremely easy, 185 (53%) very easy, and 46 (13%) moderately easy, with only three reporting any difficulty. In multivariable analysis, lower ease-of-use ratings were more frequent among participants aged ≥35 years (Table S5). Willingness to reuse HIVST was also high (309/351, 88%) and was strongly associated with perceived ease of use; reuse intention was higher among recipients and those without Medicare coverage (Table S6). Willingness to use HIVST as an interim test between clinic visits was greater among participants not recently tested and among those reporting lapsed or on-demand PrEP use (Table S7).

In exploratory analyses of test promoter performance, the mean number of HIVST kits distributed per promoter was 2.69 (maximum three per promoter); 75% distributed all three kits (Fig. 3b). In multivariable analysis (Table S8), promoters whose first language was not English distributed more kits than English-first-language promoters (1.20, 1.11–1.30; p < 0.001). Greater LGBTQ+ social connectedness was also associated with higher distribution: promoters spending “some” (1.28, 1.10–1.49; p = 0.001) or “most” (1.17, 1.01–1.36; p = 0.043) of their social time with LGBTQ+ peers distributed more kits than those spending little or none.

Descriptive comparisons between recruitment waves should be interpreted cautiously due to the small number of second-wave promoters (n = 8). Nevertheless, a markedly higher proportion of second-wave test promoters were Medicare-ineligible compared with first-wave promoters (87.5% vs 29.7%; p = 0.002; Table S9), while age distributions were broadly similar between groups. Among recipients, second-wave participants were younger, more likely to be Medicare-ineligible, and more likely to report longer intervals since last HIV testing, including never having tested, than first-wave recipients (Table S10).

Two invalid HIVST results were reported and attributed to user error; replacement kits were issued. Participants reporting reactive HIVST results received standardised instructions for confirmatory testing and referral to local sexual health services. Two reactive results were reported: one occurred in an individual with a previously diagnosed HIV infection who was already engaged in care, and the other was referred for confirmatory testing. No adverse events related to HIVST use or study participation were reported.

## Discussion

This demonstration study shows that social network-based distribution of HIVST is feasible, acceptable, and rapidly adopted among MSM in Australian settings, particularly in jurisdictions with established HIV prevention infrastructure. Our findings align with US studies showing that peer and network-based distribution of HIVST is feasible in high-income settings and can leverage existing prevention engagement (including among PrEP users) to extend testing access through social and sexual networks.^16^^,^^17^ Across both distribution waves, 88% of kits intended for recipients (260/297) were survey-verified as used, indicating strong engagement and trust in peer-mediated approaches. Incorporating time-to-use indicators—tests use within 24 h and within 7 days—provided a more precise measure of responsiveness than the “ever used” metrices that dominate HIVST research.^21^, ^22^, ^23^ More than two-thirds of recipients tested within a week and almost half within 24 h, demonstrating that HIVST can translate intention into immediate action when embedded in trusted social networks.

The model engaged MSM who experience specific barrier within conventional testing pathways, including individuals without government-subsidised healthcare, those with longer intervals since their last HIV test, and those with limited engagement in LGBTQ+ social networks. Although most participants reported prior HIV testing and PrEP use, high post-test survey completion, alongside consistently strong usability ratings and willingness to reuse the kit, further supports the feasibility and acceptability of network-based distribution. Together, these findings suggest that integrating HIVST into social networks can effectively complement clinic-based services in high-income settings where access barriers persist.

Our findings also point to a potentially important implementation insight. Individuals with stronger LGBTQ+ community connectedness were more effective as test promoters, whereas those with weaker social ties were more likely to use HIVST promptly once reached. This apparent complementarity suggests that differentiating recruitment and distribution roles within network-based HIVST programmes—by engaging highly connected individuals as distributors while prioritising outreach to less socially connected MSM as recipients—may help maximise both reach and timely uptake. Such an approach could strengthen equity by leveraging social capital to extend testing access to individuals who are less embedded in LGBTQ+ communities and experience barriers to routine engagement with clinic-based or community-led services. Future studies should formally evaluate role-differentiated network strategies.

At the same time, network-based HIVST distribution raises important equity considerations. Diffusion within social networks may be biased toward more socially connected groups, creating an inherent trade-off between efficiency and inclusivity. While leveraging well-connected networks can enhance reach and scale, it may also risk concentrating distribution within larger or denser social clusters, potentially limiting access for more socially isolated individuals. Addressing this tension will require deliberate programme design, including the use of complementary distribution channels, such as online ordering, clinic-based referral, or targeted community outreach, to ensure that network-based approaches expand, rather than inadvertently narrow, equitable access to HIV testing.

The emergence of a second distribution wave, in which recipients became promoters, expanded reach into additional networks and facilitated uptake among secondary recipients. This cascading diffusion, analogous to respondent-driven sampling, highlights the added value of multi-wave approaches for engaging socially diffuse subgroups. Even with a modest number of second-wave promoters, the model extended to Medicare-ineligible men with longer testing lapses, mirroring diffusion patterns observed in other network-based interventions.^14^^,^^24^^,^^25^

This study extends evidence from sub-Saharan Africa and Asia into to a high-income, multicultural context, demonstrating that social network-based distribution of HIVST can operate effectively within universal healthcare systems where social and structural inequities continue to limit testing access.^7^^,^^23^^,^^26^, ^27^, ^28^ Although a subset of MSM in Australia remain untested or test infrequently,^29^ only a small proportion of participants in this demonstration study (3%) reported never having tested for HIV. As such, the primary contribution of this approach in this context relates to feasibility, acceptability, and timely testing rather than identification of previously undiagnosed HIV infection. The rapid uptake and limited onward diffusion observed indicate that social networks can support responsive and convenient testing within existing prevention ecosystems, and highlight how strategies developed in resource-limited settings may be adapted to complement established testing services in high-income health systems.

Rapid usage among participants with longer testing gaps indicates that network-based HIVST may be particularly effective for re-engaging lapsed testers. Timely self-testing has meaningful implications for prevention; delays between receiving a test and using it contribute to prolong periods of undiagnosed HIV and, consequently increased community viral load.^30^ The high proportion of recipients who tested immediately—almost half within 24 h—suggests strong confidence in HIVST and minimal barriers to action. These patterns are consistent with social influence and normative activation processes, whereby endorsement or modelling of behaviour by trusted peers strengthens perceived norms and facilitates timely uptake.^31^^,^^32^

At the same time, delayed use of HIVST should not necessarily be interpreted as reduced acceptability or engagement. A substantial proportion of participants, particularly test promoters, reported HIV testing within the previous three months and were engaged in PrEP care. For individuals who had tested recently or were accessing routine testing through PrEP services, deferring HIVST use until a more clinically appropriate time point may reflect intentional and appropriate use. Time-to-use outcomes should therefore be interpreted in the context of recent testing history and prevention engagement.

Usability and acceptability were high across the sample, supporting the feasibility of network-based distribution. Most participants found the tests easy to use and were willing to use HIVST again, including Medicare-ineligible individuals and those with longer intervals since their last HIV test—groups that often face structural or financial barriers to clinic-based testing. Compatibility with existing testing routines was also strong: many participants identified HIVST as suitable for use between clinic visits, pointing to opportunities to integrate HIVST alongside PrEP refills, home STI testing, or for rapid testing following PrEP interruptions.^8^ Slightly lower ease-of-use ratings among participants aged ≥35 years; however, overall usability remained high across all age groups. These findings suggest that modest usability refinements, such as larger fonts, clearer pictograms, and simple digital guides, could further enhance accessibility across diverse age, literacy, and language groups.

Our findings have direct implications for policy and implementation. Embedding network-based HIVST within PrEP, sexual health, and primary care programmes may enhance convenience and flexibility of testing, particularly for individuals who face financial, cultural, or logistical barriers within clinic-based pathways. Light-touch prompts, such as brief reminder emails or text messages sent shortly after distribution may further improve uptake without compromising autonomy. These prompts can gently remind recipients to use the self-test or complete follow-up activities while avoiding the intensity of active outreach or case management. Clear linkage pathways (e.g., peer navigation, multilingual helplines, or app-based guidance) remain essential to ensure that rapid self-testing leads to confirmatory diagnosis, timely ART initiation, and seamless PrEP continuation. Integrating network-based HIVST into existing prevention infrastructures could support more responsive testing practices while reducing reliance on clinic-based services.

At a systems level, these findings highlight the value of social networks as both delivery channels and mechanisms of behaviour change. Social network-based distribution of HIVST decentralises prevention by positioning peers as users, promoters, and facilitators, shifting decision-making from institutions to communities. This approach enhances trust, cultural resonance, and autonomy,^33^ and aligns with WHO's self-care framework and the UNAIDS agenda on community-led responses.^34^^,^^35^ Ensuring equity will require intentional programme design, including culturally and linguistically tailored materials and monitoring systems capable of identifying whether diffusion patterns inadvertently reinforce existing disparities.

Compared with online HIVST distribution models, which can reach large numbers of individuals rapidly, the social network-based approach evaluated here operated at a smaller scale, distributing relatively few kits per day. Network-based distribution should therefore be viewed as a complementary strategy that may enhance reach to individuals less likely to engage with online or clinic-based services, rather than as a substitute for high-throughput online programmes.

In practice, progress in HIV prevention requires both formal research studies and timely demonstration projects that generate real-world evidence. While controlled research designs are essential for establishing efficacy and causal inference, they can be slow to complete and may not fully capture implementation realities. Demonstration projects such as this one play a complementary role by providing pragmatic data on feasibility, acceptability, and uptake in routine settings, helping inform policy and programme decisions while more definitive studies are underway.

This study has limitations. Participants were self-selected and may have been more motivated to test than MSM in the wider community, limiting generalisability. The study population was characterised by high baseline engagement with HIV prevention services, with most participants reporting prior HIV testing and current PrEP use. Consequently, the population reached may not fully represent MSM who have never tested for HIV or who access testing infrequently, and findings should be interpreted primarily in terms of feasibility and acceptability rather than optimal targeting or case-finding. Evidence of onward diffusion should be interpreted cautiously, as the second-wave sample was small, limiting inferences about multi-wave dynamics. All outcomes were self-reported, introducing potential recall or social desirability bias. HIV self-test results were self-reported, and photographs of completed tests were not collected, limiting independent assessment of correct test use and result interpretation. However, evidence from recent research suggests that MSM are generally able to correctly interpret and report HIV self-test results, which may mitigate concerns regarding misclassification due to self-report.^36^ Nonetheless, the absence of objective verification in the present study remains a limitation. Although recruitment was open nationally, participation was concentrated in Victoria and New South Wales, with limited representation from other states and territories; findings may therefore primarily reflect settings with more established HIV prevention infrastructure. Further evaluation in jurisdictions with smaller or more dispersed MSM populations is needed to assess scalability and generalisability across the broader Australian context. Information on Aboriginal and Torres Strait Islander identity was not collected, limiting assessment of representativeness and equity implications for Indigenous peoples in Australia. Modest monetary incentives were provided to test promoters for survey completion, which may have contributed to high levels of survey verification and could overestimate uptake in the absence of financial compensation. Clustering by social network was not explicitly modelled; however, networks were small with minimal overlap, limiting the potential impact of intra-network correlation. Finally, longer-term outcomes such as repeat testing and sustained distribution were not assessed, and the absence of a comparison arm limits causal inference.

Future research should compare network-based HIVST with pharmacy, digital, and clinic-based models, focusing on time to test, case-finding yield, and linkage outcomes. Network analytics could identify promoter characteristics that optimise diffusion and inform targeted resupply strategies. Studies should prioritise individuals who have never tested or test infrequently to assess reach among those at greatest risk of undiagnosed HIV. Mixed-methods research and social network modelling could further elucidate how trust, network density, and social capital shape diffusion. Economic evaluations incorporating equity metrics are needed to ensure that scale-up reduces, rather than entrenches, disparities in testing access.

In conclusion, social network-based distribution of HIVST was feasible, acceptable, and achieved rapid uptake among MSM in Australia. In a context of high baseline HIV testing coverage and widespread PrEP use, the primary contribution of this approach lies in enhancing the convenience, timeliness, and flexibility of testing within existing prevention systems rather than in identifying large numbers of previously undiagnosed infections. By leveraging peer networks, this model complements clinic-based and online testing strategies and offers a pragmatic mechanism to support responsive testing in real-world settings. Future implementations that intentionally prioritise under-tested populations are needed to assess the potential of network-based HIV self-testing to reduce inequities in access to HIV testing and to strengthen its role within comprehensive HIV prevention programmes.

## Contributors

YZ and JJO had full access to all the data in the study and verified the underlying data. YZ contributed to conceptualisation, recruitment, data collection, formal analysis, and writing of the original draft, and participated in reviewing and editing the manuscript. JJO contributed to conceptualisation, supervision, and reviewing and editing the manuscript. CCJ, MT, JAD, EPFC, WT and MS contributed to reviewing and editing the manuscript. All authors were responsible for the decision to submit the manuscript for publication.

## Data sharing statement

The data will be shared on reasonable request to the corresponding author.

## Declaration of interests

MT reports consulting fees from Gilead Sciences Ltd. EC reports an investigator grant from MSD paid to his institution and honoraria from MSD for consultation related to human papillomavirus. All authors declare no other competing interests. The findings and conclusions in this report are those of the authors and do not necessarily represent the official position of the WHO.
