# Supplementary material for: Social network distribution of HIV self-tests among MSM in Australia: a prospective, non-randomised trial
Source: Lancet Reg Health West Pac. 2026 Mar 24;69:101839. doi: 10.1016/j.lanwpc.2026.101839 (PMC13045671; doi:10.1016/j.lanwpc.2026.101839)
Supplement: Supplementary Figures and Tables [file mmc1.pdf]

Table of Contents

Table S1: HIVST usage from time of kit receipt by role (test promoter vs recipient) (N=351) *2*

Table S2: Multivariable Poisson regression of factors associated with HIVST use within 24 hours (N=351) *3*

Table S3: Recipient-only Poisson regression of HIVST usage within 7 days (N=260) *5*

Table S4: Recipient-only Poisson regression of HIVST usage within 24 hours (N=260) *7*

Figure S1: Perceived ease of HIVST use by participant role (N=351) *9*

Figure S2: Likelihood of using an HIVST kit again by participant role (N=351) *9*

Figure S3: Willingness to use an HIVST kit as interim test between clinic visits by participant role (N=351) *10*

Table S5: Multivariable ordinal logistic regression of perceived ease of HIVST use (recategorised: not easy, moderately easy, very easy) (N=351) *11*

Table S6: Multivariable ordinal logistic regression of likelihood to use an HIVST kit again (recategorised: not likely, moderately likely, very likely) (N=351) *13*

Table S7: Multivariable Poisson regression for willingness to use an HIVST kit as interim test between clinic visits (N=351) *15*

Table S8: Multivariable Poisson regression showing predictors of strong test promoter performance (measured by proportion of HIVST kits distributed) (N=91) *17*

Table S9: Comparison of test promoter characteristics between Wave 1 and Wave 2 (N=99) *19*

Table S10: Comparison of recipient characteristics between Wave 1 and Wave 2 (N=260) *21*

Table S11: CONSORT checklist *23*

Table S12: TREND checklist *25*

Table S1: HIVST usage from time of kit receipt by role (test promoter vs recipient) (N=351)

	Recipient (n=260)	Test promoter (n=91)	Total
**0–12 hours**	76	15	91
**>12–24 hours**	71	19	90
**>24 hours–7 days**	75	24	99
**More than 7 days**	38	33	71
**Total**	260	91	351

N=351

Table S2: Multivariable Poisson regression of factors associated with HIVST use within 24 hours (N=351)

	HIVST use within 24 hours, n/N (%)	Univariate		Multivariable	
		Risk ratio	p-value	Adjusted risk ratio	p-value
**Role**					
Test promoter	34/91 (37.4)	Ref	Ref	Ref	Ref
Recipient	147/260 (56.5)	1.51 (1.14–2.02)	0.005	1.21 (0.92–1.58)	0.175
**Age (years)**					
18–24	36/53 (67.9)	Ref	Ref	NA	NA
25–34	88/169 (52.1)	0.77 (0.61–0.97)	0.027	NA	NA
≥35	57/129 (44.2)	0.65 (0.50–0.85)	0.002	NA	NA
**State**					
Australian Capital Territory	3/3 (100.0)	Ref	Ref	Ref	Ref
New South Wales	26/49 (53.1)	0.53 (0.41–0.69)	<0.001	0.56 (0.40–0.78)	0.001
Queensland	11/12 (91.7)	0.92 (0.77–1.09)	0.318	0.94 (0.67–1.31)	0.701
South Australia	2/4 (50.0)	0.50 (0.19–1.33)	0.166	0.56 (0.28–1.14)	0.109
Victoria	134/277 (48.4)	0.48 (0.43–0.55)	<0.001	0.59 (0.45–0.76)	<0.001
Western Australia	5/6 (83.3)	0.83 (0.58–1.19)	0.319	0.95 (0.67–1.34)	0.770
**Medicare status***					
Medicare-eligible	88/203 (43.3)	Ref	Ref	Ref	Ref
Medicare-ineligible	93/148 (62.8)	1.45 (1.19–1.77)	<0.001	1.36 (1.11–1.67)	0.004
**First language other than English**					
No	50/83 (60.2)	Ref	Ref	Ref	Ref
Yes	131/268 (48.9)	0.81 (0.66–1.00)	0.055	0.72 (0.59–0.88)	0.001
**HIV testing recency**					
<3 months ago	32/113 (28.3)	Ref	Ref	Ref	Ref
3–6 months ago	43/87 (49.4)	1.74 (1.21–2.50)	0.003	1.62 (1.13–2.31)	0.008
7–12 months ago	53/76 (69.7)	2.46 (1.77–3.42)	<0.001	2.19 (1.57–3.04)	<0.001
Over 12 months ago	46/68 (67.6)	2.39 (1.71–3.35)	<0.001	2.02 (1.41–2.88)	<0.001
Never tested for HIV	7/7 (100.0)	3.53 (2.63–4.74)	<0.001	2.59 (1.79–3.74)	<0.001
**Education level attained**					
Bachelor	98/172 (57.0)	Ref	Ref	NA	NA
High school	19/26 (73.1)	1.28 (0.98–1.68)	0.068	NA	NA
Certificate/Diploma	15/36 (41.7)	0.73 (0.49–1.10)	0.133	NA	NA

Postgraduate	49/116 (42.2)	0.74 (0.58–0.95)	0.019	NA	NA
**Community connectedness** *(How much of your social time do you spend with LGBTQ+ friends or community?)*					
Almost all of the time	21/39 (53.8)	Ref	Ref	NA	NA
Most of the time	36/103 (35.0)	0.65 (0.44–0.96)	0.031	NA	NA
Some of the time	76/140 (54.3)	1.01 (0.73–1.40)	0.961	NA	NA
A little of the time	43/63 (68.3)	1.27 (0.91–1.77)	0.167	NA	NA
None of the time	5/6 (83.3)	1.55 (0.98–2.46)	0.064	NA	NA
**PrEP use**					
Daily PrEP	38/88 (43.2)	Ref	Ref	NA	NA
On demand PrEP	63/134 (47.0)	1.09 (0.81–1.47)	0.578	NA	NA
Lapsed**	22/33 (66.7)	1.54 (1.10–2.17)	0.012	NA	NA
Never taken PrEP	58/96 (60.4)	1.40 (1.05–1.87)	0.023	NA	NA
**Number of sexual partners in last 12 months**					
0–1	35/51 (68.6)	Ref	Ref	NA	NA
2–5	67/111 (60.4)	0.88 (0.69–1.12)	0.293	NA	NA
6–10	43/79 (54.4)	0.79 (0.60–1.04)	0.098	NA	NA
>10	36/110 (32.7)	0.48 (0.34–0.66)	<0.001	NA	NA

HIV, human immunodeficiency virus; LGBTQ+, lesbian, gay, bisexual, transgender and queer people; NA, not applicable; PrEP, pre-exposure prophylaxis; Ref, reference level

* Australia's Medicare is a publicly funded universal healthcare system that provides free or subsidised access to medical services, hospital care, and prescription medications for Australian citizens and permanent residents

** Participants self-identifying as having previously taken PrEP but not currently using it were categorised as "lapsed".

Table S3: Recipient-only Poisson regression of HIVST usage within 7 days (N=260)

	Univariate		Multivariable	
	**Risk ratio**	**p-value**	**Adjusted risk ratio**	**p-value**
**Age (years)**				
18–24	Ref	Ref	Ref	Ref
25–34	0.99 (0.90–1.10)	0.924	1.13 (1.02–1.25)	0.020
≥35	0.80 (0.69–0.94)	0.007	0.97 (0.83–1.14)	0.707
**State**				
Australian Capital Territory	Ref	Ref	NA	NA
New South Wales	0.87 (0.77–0.98)	0.206	NA	NA
Queensland	1.00 (1.00–1.00)	1	NA	NA
South Australia	0.67 (0.30–1.48)	0.322	NA	NA
Victoria	0.84 (0.79–0.89)	<0.001	NA	NA
Western Australia	1.00 (1.00–1.00)	1	NA	NA
**Medicare status***				
Medicare-eligible	Ref	Ref	Ref	Ref
Medicare-ineligible	1.26 (1.14–1.39)	<0.001	1.18 (1.07–1.30)	0.001
**First language other than English**				
No	Ref	Ref	NA	NA
Yes	1.07 (0.94–1.23)	0.309	NA	NA
**HIV testing recency**				
<3 months ago	Ref	Ref	Ref	Ref
3–6 months ago	1.11 (0.91–1.34)	0.303	1.05 (0.88–1.26)	0.581
7–12 months ago	1.33 (1.14–1.56)	<0.001	1.27 (1.10–1.48)	0.002
Over 12 months ago	1.26 (1.06–1.50)	<0.001	1.15 (0.98–1.35)	0.081
Never tested for HIV	1.38 (1.18–1.61)	0.008	1.20 (1.01–1.42)	0.041
**Education level attained**				
Bachelor	Ref	Ref	NA	NA
High school	1.04 (0.89–1.21)	0.657	NA	NA
Certificate/Diploma	0.88 (0.72–1.07)	0.198	NA	NA
Postgraduate	0.83 (0.72–0.94)	0.005	NA	NA
**Community connectedness** *(How much of your social time do you spend with LGBTQ+ friends or community?)*				
Almost all of the time	Ref	Ref	Ref	Ref
Most of the time	1.08 (0.84–1.39)	0.563	1.17 (0.93–1.49)	0.187

Some of the time	1.24 (0.99–1.56)	0.058	1.26 (1.02–1.55)	0.032
A little of the time	1.41 (1.14–1.76)	0.002	1.42 (1.16–1.75)	0.001
None of the time	1.42 (1.14–1.76)	0.002	1.41 (1.12–1.78)	0.004
**PrEP use**				
Daily PrEP	Ref	Ref	NA	NA
On demand PrEP	1.01 (0.86–1.19)	0.917	NA	NA
Lapsed**	1.12 (0.93–1.33)	0.232	NA	NA
Never taken PrEP	1.15 (1.00–1.33)	0.055	NA	NA
**Number of sexual partners in last 12 months**				
0–1	Ref	Ref	NA	NA
2–5	1.03 (0.90–1.18)	0.684	NA	NA
6–10	1.01 (0.84–1.17)	0.834	NA	NA
>10	0.98 (0.78–1.08)	0.298	NA	NA

HIV, human immunodeficiency virus; LGBTQ+, lesbian, gay, bisexual, transgender and queer people; NA, not applicable; PrEP, pre-exposure prophylaxis; Ref, reference level
* Australia’s Medicare is a publicly funded universal healthcare system that provides free or subsidised access to medical services, hospital care, and prescription medications for Australian citizens and permanent residents
** Participants self-identifying as having previously taken PrEP but not currently using it were categorised as “lapsed”.

Table S4: Recipient-only Poisson regression of HIVST usage within 24 hours (N=260)

	Univariate		Multivariable	
	**Risk ratio**	**p-value**	**Risk ratio**	**p-value**
**Age (years)**				
18–24	Ref	Ref	NA	NA
25–34	0.82 (0.65–1.05)	0.115	NA	NA
≥35	0.69 (0.52–0.93)	0.015	NA	NA
**State**				
Australian Capital Territory	Ref	Ref	Ref	Ref
New South Wales	0.61 (0.47–0.78)	<0.001	0.61 (0.41–0.90)	0.014
Queensland	0.89 (0.71–1.12)	0.319	0.83 (0.54–1.27)	0.385
South Australia	0.67 (0.30–1.49)	0.322	0.57 (0.29–1.11)	0.096
Victoria	0.53 (0.47–0.61)	<0.001	0.59 (0.41–0.84)	0.004
Western Australia	0.80 (0.52–1.24)	0.319	0.86 (0.53–1.38)	0.529
**Medicare status***				
Medicare-eligible	Ref	Ref	Ref	Ref
Medicare-ineligible	1.45 (1.17–1.80)	0.001	1.31 (1.03–1.66)	0.025
**First language other than English**				
No	Ref	Ref	Ref	Ref
Yes	0.87 (0.68–1.10)	0.229	NA	NA
**HIV testing recency**				
<3 months ago	Ref	Ref	Ref	Ref
3–6 months ago	1.46 (0.96–2.22)	0.081	1.30 (0.87–1.94)	0.199
7–12 months ago	2.13 (1.45–3.12)	<0.001	1.76 (1.21–2.56)	0.003
Over 12 months ago	2.00 (1.35–2.96)	<0.001	1.41 (0.92–2.14)	0.111
Never tested for HIV	2.95 (2.08–4.18)	<0.001	1.79 (1.20–2.68)	0.005
**Education level attained**				
Bachelor	Ref	Ref	Ref	Ref
High school	1.28 (0.98–1.68)	0.068	1.10 (0.83–1.46)	0.526
Certificate/Diploma	0.73 (0.49–1.10)	0.133	0.80 (0.55–1.17)	0.247
Postgraduate	0.74 (0.58–0.95)	0.019	0.86 (0.68–1.12)	0.266
**Community connectedness** *(How much of your social time do you spend with LGBTQ+ friends or community?)*				
Almost all of the time	Ref	Ref	NA	NA
Most of the time	0.75 (0.50–1.13)	0.167	NA	NA

Some of the time	1.06 (0.76–1.49)	0.725	NA	NA
A little of the time	1.20 (0.85–1.72)	0.304	NA	NA
None of the time	1.49 (0.93–2.38)	0.094	NA	NA
**PrEP use**				
Daily PrEP	Ref	Ref	NA	NA
On demand PrEP	1.18 (0.83–1.67)	0.355	NA	NA
Lapsed**	1.62 (1.12–2.35)	0.010	NA	NA
Never taken PrEP	1.43 (1.02–2.00)	0.038	NA	NA
**Number of sexual partners in last 12 months**				
0–1	Ref	Ref	Ref	Ref
2–5	0.91 (0.71–1.17)	0.478	0.91 (0.69–1.19)	0.483
6–10	0.89 (0.67–1.17)	0.393	0.92 (0.69–1.24)	0.596
>10	0.50 (0.34–0.73)	<0.001	0.63 (0.42–0.95)	0.026

HIV, human immunodeficiency virus; LGBTQ+, lesbian, gay, bisexual, transgender and queer people; NA, not applicable; PrEP, pre-exposure prophylaxis; Ref, reference level
* Australia's Medicare is a publicly funded universal healthcare system that provides free or subsidised access to medical services, hospital care, and prescription medications for Australian citizens and permanent residents
** Participants self-identifying as having previously taken PrEP but not currently using it were categorised as "lapsed".

Figure S1: Perceived ease of HIVST use by participant role (N=351)

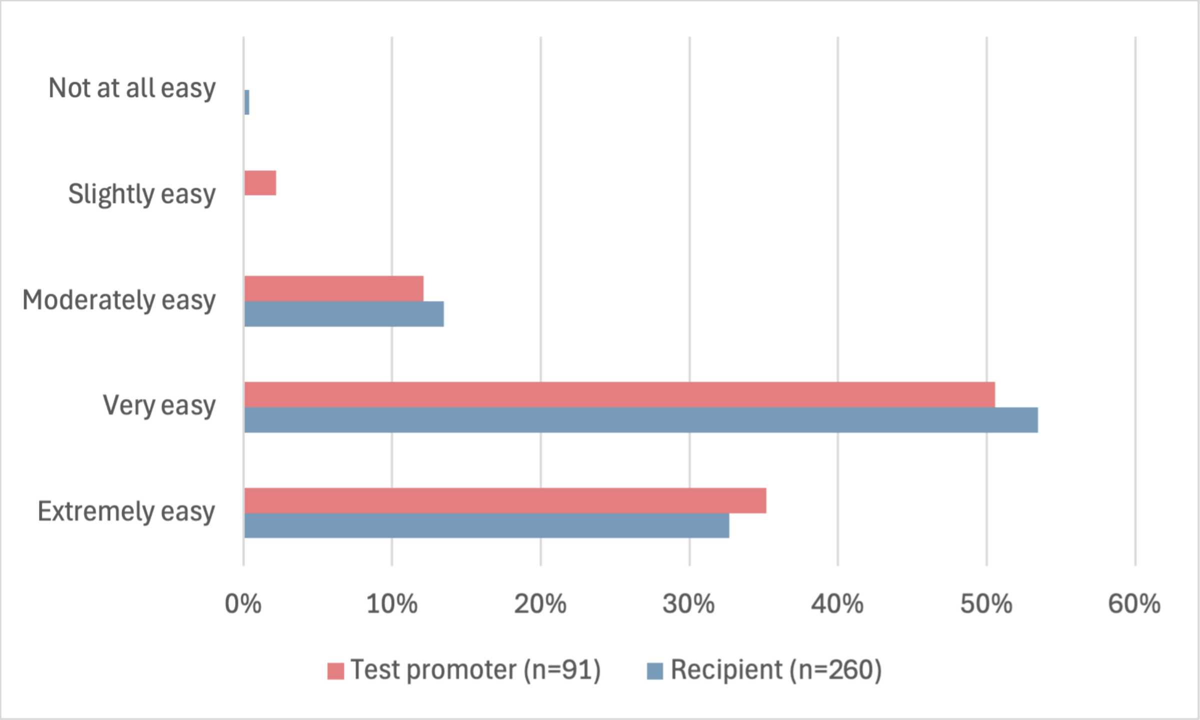

Figure S2: Likelihood of using an HIVST kit again by participant role (N=351)

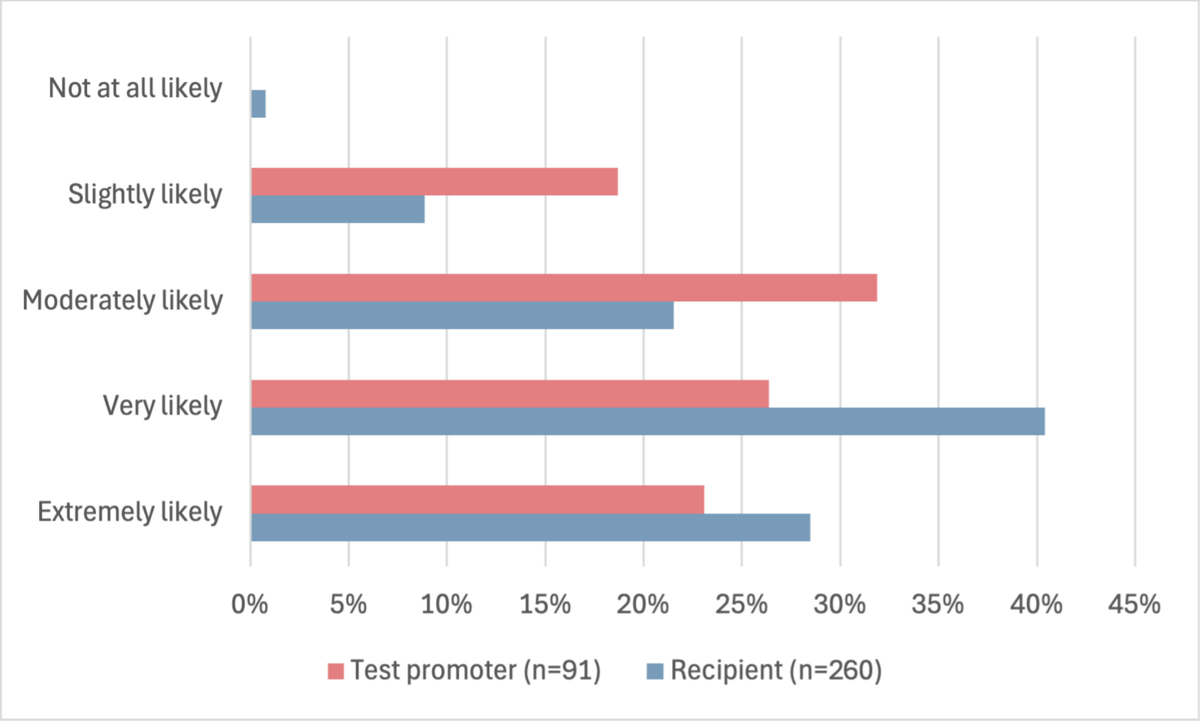

Figure S3: Willingness to use an HIVST kit as interim test between clinic visits by participant role (N=351)

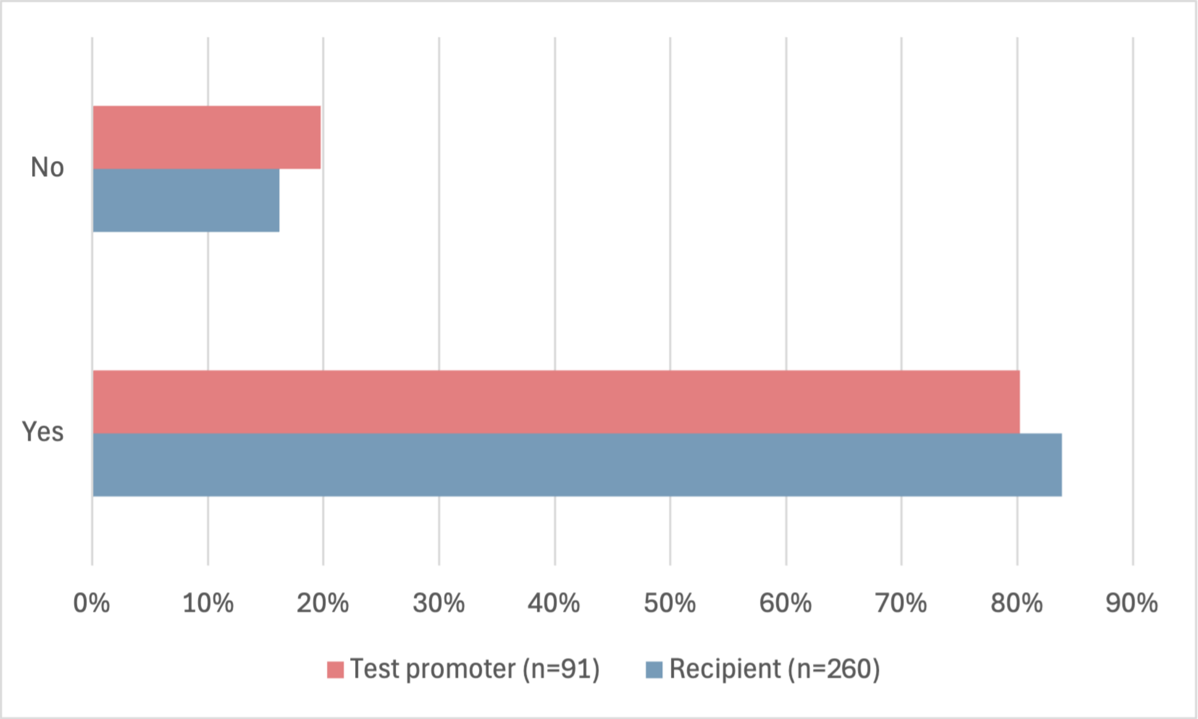

Table S5: Multivariable ordinal logistic regression of perceived ease of HIVST use (recategorised: not easy, moderately easy, very easy) (N=351)

	Univariate		Multivariable	
	**Odds ratio**	**p-value**	**Adjusted odds ratio**	**p-value**
**Role**				
Test promoter	Ref	Ref	NA	NA
Recipient	1.06 (0.53–2.12)	0.871	NA	NA
**Age (years)**				
18–24	Ref	Ref	Ref	Ref
25–34	0.38 (0.11–1.31)	0.125	0.26 (0.06–1.10)	0.066
≥35	0.28 (0.08–0.96)	0.043	0.21 (0.05–0.94)	0.041
**State**				
New South Wales	Ref	Ref	NA	NA
Australian Capital Territory*	NA	NA	NA	NA
Queensland	0.89 (0.08–10.42)	0.926	NA	NA
South Australia*	NA	NA	NA	NA
Victoria	0.47 (0.16–1.37)	0.166	NA	NA
Western Australia*	NA	NA	NA	NA
**Medicare status****				
Medicare-eligible	Ref	Ref	NA	NA
Medicare-ineligible	1.06 (0.57–1.95)	0.864	NA	NA
**First language other than English**				
No	Ref	Ref	Ref	Ref
Yes	0.93 (0.45–1.92)	0.845	0.84 (0.41–1.72)	0.630
**HIV testing recency**				
<3 months ago	Ref	Ref	NA	NA
3–6 months ago	0.53 (0.24–1.19)	0.123	NA	NA
7–12 months ago	0.71 (0.30–1.70)	0.445	NA	NA
Over 12 months ago	0.69 (0.28–1.70)	0.415	NA	NA
Never tested for HIV*	NA	NA	NA	NA
**Education level attained**				
Bachelor	Ref	Ref	Ref	Ref
High school	0.66 (0.21–2.11)	0.482	0.39 (0.11–1.34)	0.135
Certificate/Diploma	0.25 (0.11–0.57)	0.001	0.26 (0.11–1.34)	0.002

Postgraduate	0.87 (0.41–1.83)	0.713	1.02 (0.46–2.26)	0.959
**Community connectedness** *(How much of your social time do you spend with LGBTQ+ friends or community?)*				
Almost all of the time	Ref	Ref	NA	NA
Most of the time	1.82 (0.66–5.03)	0.250	NA	NA
Some of the time	1.56 (0.60–4.05)	0.360	NA	NA
A little of the time	0.74 (0.27–2.02)	0.556	NA	NA
None of the time*	NA	NA	NA	NA
**PrEP use**				
Daily PrEP	Ref	Ref	NA	NA
On demand PrEP	2.38 (1.10–5.16)	0.028	NA	NA
Lapsed***	1.17 (0.43–3.22)	0.761	NA	NA
Never taken PrEP	1.79 (0.81–3.98)	0.153	NA	NA
**Number of sexual partners in last 12 months**				
0–1	Ref	Ref	NA	NA
2–5	1.27 (0.50–3.24)	0.613	NA	NA
6–10	1.13 (0.42–3.03)	0.806	NA	NA
0–1	1.08 (0.43–2.70)	0.869	NA	NA

HIV, human immunodeficiency virus; LGBTQ+, lesbian, gay, bisexual, transgender and queer people; NA, not applicable; PrEP, pre-exposure prophylaxis; Ref, reference level

* Excluded from regression models due to insufficient sample size leading to unstable estimates.

** Australia's Medicare is a publicly funded universal healthcare system that provides free or subsidised access to medical services, hospital care, and prescription medications for Australian citizens and permanent residents.

*** Participants self-identifying as having previously taken PrEP but not currently using it were categorised as "lapsed".

Table S6: Multivariable ordinal logistic regression of likelihood to use an HIVST kit again (recategorised: not likely, moderately likely, very likely) (N=351)

	Univariate		Multivariable	
	**Odds ratio**	**p-value**	**Adjusted odds ratio**	**p-value**
**Role**				
Test promoter	Ref	Ref	Ref	Ref
Recipient	2.24 (1.40–3.57)	0.001	1.75 (1.02–3.03)	0.044
**Age**				
18–24	Ref	Ref	Ref	Ref
25–34	0.72 (0.36–1.47)	0.368	1.03 (0.51–2.11)	0.930
≥35	0.37 (0.18–0.74)	0.005	0.77 (0.36–1.65)	0.500
**State**				
Australian Capital Territory	Ref	Ref	NA	NA
New South Wales	0.55 (0.07–4.49)	0.574	NA	NA
Queensland	0.50 (0.05–5.62)	0.578	NA	NA
South Australia*	NA	NA	NA	NA
Victoria	0.80 (0.10–6.15)	0.829	NA	NA
Western Australia	1.00 (0.08–11.97)	1	NA	NA
**Medicare status****				
Medicare-eligible	Ref	Ref	Ref	Ref
Medicare-ineligible	2.66 (1.63–4.35)	<0.001	2.11 (1.23–3.61)	0.007
**First language other than English**				
No	Ref	Ref	Ref	Ref
Yes	1.58 (0.99–2.53)	0.056	1.17 (0.69–1.99)	0.553
**HIV testing recency**				
<3 months ago	Ref	Ref	NA	NA
3–6 months ago	0.99 (0.55–1.77)	0.978	NA	NA
7–12 months ago	0.98 (0.56–1.73)	0.957	NA	NA
Over 12 months ago	1.31 (0.68–2.54)	0.415	NA	NA
Never tested for HIV	3.79 (0.48–29.71)	0.204	NA	NA
**Education level attained**				
Bachelor	Ref	Ref	NA	NA
High school	0.83 (0.32–2.13)	0.698	NA	NA
Certificate/Diploma	1.07 (0.63–2.16)	0.852	NA	NA
Postgraduate	0.70 (0.44–1.14)	0.151	NA	NA

**Community connectedness** *(How much of your social time do you spend with LGBTQ+ friends or community?)*				
Almost all of the time	Ref	Ref	NA	NA
Most of the time	0.79 (0.34–1.82)	0.574	NA	NA
Some of the time	0.91 (0.41–2.03)	0.812	NA	NA
A little of the time	0.72 (0.31–1.69)	0.451	NA	NA
None of the time	2.55 (0.30–21.94)	0.394	NA	NA
**PrEP use**				
Daily PrEP	Ref	Ref	Ref	Ref
On demand PrEP	1.76 (1.03–3.00)	0.039	1.32 (0.73–2.36)	0.348
Lapsed***	1.83 (0.88–3.78)	0.104	1.32 (0.59–2.98)	0.492
Never taken PrEP	2.29 (1.25–4.22)	0.008	1.33 (0.69–2.56)	0.389
**Number of sexual partners in last 12 months**				
0–1	Ref	Ref	NA	NA
2–5	0.83 (0.41–1.67)	0.594	NA	NA
6–10	0.77 (0.36–1.63)	0.494	NA	NA
>10	0.57 (0.28–1.17)	0.124	NA	NA
**Ease of HIV self-test use**				
Not easy	Ref	Ref	Ref	Ref
Moderately easy	2.62 (0.76–9.06)	0.128	2.69 (0.37–19.59)	0.328
Very easy	8.80 (2.57–30.13)	0.001	8.73 (1.20–63.63)	0.032

HIV, human immunodeficiency virus; LGBTQ+, lesbian, gay, bisexual, transgender and queer people; NA, not applicable; PrEP, pre-exposure prophylaxis; Ref, reference level

* Excluded from regression models due to insufficient sample size leading to unstable estimates.

** Australia's Medicare is a publicly funded universal healthcare system that provides free or subsidised access to medical services, hospital care, and prescription medications for Australian citizens and permanent residents.

*** Participants self-identifying as having previously taken PrEP but not currently using it were categorised as "lapsed".

Table S7: Multivariable Poisson regression for willingness to use an HIVST kit as interim test between clinic visits (N=351)

	Univariate		Multivariable	
	**Risk ratio**	**p-value**	**Adjusted risk ratio**	**p-value**
**Role**				
Test promoter	Ref	Ref	Ref	Ref
Recipient	1.05 (0.93–1.17)	0.452	NA	NA
**Age (years)**				
18–24	Ref	Ref	NA	NA
25–34	0.96 (0.86–1.08)	0.496	NA	NA
≥35	0.87 (0.76–1.00)	0.049	NA	NA
**State**				
Australian Capital Territory	Ref	Ref	NA	NA
New South Wales	0.88 (0.79–0.97)	0.015	NA	NA
Queensland	0.92 (0.77–1.09)	0.318	NA	NA
South Australia	0.75 (0.43–1.32)	0.32	NA	NA
Victoria	0.82 (0.77–0.86)	<0.001	NA	NA
Western Australia	0.83 (0.58–1.19)	0.319	NA	NA
**Medicare status***				
Medicare-eligible	Ref	Ref	Ref	Ref
Medicare-ineligible	1.11 (1.01–1.21)	0.030	1.05 (0.95–1.16)	0.329
**First language other than English**				
No	Ref	Ref	NA	NA
Yes	0.94 (0.85–1.04)	0.249	NA	NA
**HIV testing recency**				
<3 months ago	Ref	Ref	Ref	Ref
3–6 months ago	1.28 (1.11–1.48)	0.001	1.24 (1.08–1.42)	0.002
7–12 months ago	1.28 (1.11–1.48)	0.001	1.18 (1.02–1.37)	0.023
Over 12 months ago	1.24 (1.07–1.45)	0.006	1.15 (1.00–1.34)	0.070
Never tested for HIV	1.43 (1.27–1.61)	<0.001	1.28 (1.12–1.45)	<0.001
**Education level attained**				
Bachelor	Ref	Ref	NA	NA
High school	1.10 (1.00–1.20)	0.061	NA	NA
Certificate/Diploma	0.79 (0.63–0.99)	0.040	NA	NA
Postgraduate	0.87 (0.78–0.98)	0.021	NA	NA
**Community connectedness** *(How much of your social time do you spend with LGBTQ+ friends or community?)*				

Almost all of the time	Ref	Ref	NA	NA
Most of the time	1.01 (0.81–1.28)	0.904	NA	NA
Some of the time	1.24 (1.01–1.53)	0.037	NA	NA
A little of the time	1.26 (1.02–1.56)	0.033	NA	NA
None of the time	1.39 (1.14–1.70)	0.001	NA	NA
**PrEP use**				
Daily PrEP	Ref	Ref	Ref	Ref
On demand PrEP	1.32 (1.12–1.56)	0.001	1.27 (1.08–1.50)	0.005
Lapsed**	1.43 (1.20–1.70)	<0.001	1.33 (1.12–1.59)	0.001
Never taken PrEP	1.34 (1.14–1.59)	0.001	1.26 (1.04–1.51)	0.016
**Number of sexual partners in last 12 months**				
0–1	Ref	Ref	NA	NA
2–5	1.02 (0.88–1.17)	0.835	NA	NA
6–10	1.07 (0.93–1.23)	0.371	NA	NA
>10	0.88 (0.75–1.04)	0.135	NA	NA

HIV, human immunodeficiency virus; LGBTQ+, lesbian, gay, bisexual, transgender and queer people; NA, not applicable; PrEP, pre-exposure prophylaxis; Ref, reference level

* Australia's Medicare is a publicly funded universal healthcare system that provides free or subsidised access to medical services, hospital care, and prescription medications for Australian citizens and permanent residents

** Participants self-identifying as having previously taken PrEP but not currently using it were categorised as "lapsed".

Table S8: Multivariable Poisson regression showing predictors of strong test promoter performance (measured by proportion of HIVST kits distributed) (N=91)

	Univariate		Multivariable	
	**Risk ratio**	**p-value**	**Adjusted risk ratio**	**p-value**
**Age (years)**				
18–24	Ref	Ref	NA	NA
25–34	0.99 (0.89–1.10)	0.877	NA	NA
≥35	0.99 (0.90–1.10)	0.923	NA	NA
**State**				
Australian Capital Territory	Ref	Ref	NA	NA
New South Wales	0.95 (0.90–1.01)	0.129	NA	NA
Queensland	0.83 (0.71–0.98)	0.026	NA	NA
South Australia	NA	NA	NA	NA
Victoria	0.92 (0.89–0.95)	<0.001	NA	NA
Western Australia	NA	NA	NA	NA
**Medicare status***				
Medicare-eligible	Ref	Ref	Ref	Ref
Medicare-ineligible	1.08 (1.02–1.14)	0.011	1.01 (0.96–1.07)	0.617
**First language other than English**				
No	Ref	Ref	Ref	Ref
Yes	1.22 (1.11–1.34)	<0.001	1.20 (1.11–1.30)	<0.001
**HIV testing recency**				
<3 months ago	Ref	Ref	Ref	Ref
3–6 months ago	0.99 (0.89–1.10)	0.828	1.04 (0.99 – 1.08)	0.107
7–12 months ago	1.02 (0.93–1.11)	0.690	0.98 (0.94 – 1.02)	0.369
Over 12 months ago	1.06 (0.99–1.13)	0.086	0.96 (0.90 – 1.02)	0.166
Never tested for HIV	NA	NA	NA	NA
**Education level attained**				
Bachelor	Ref	Ref	NA	NA
High school	0.99 (0.85–1.15)	0.881	NA	NA
Certificate/Diploma	0.94 (0.84–1.06)	0.328	NA	NA
Postgraduate	1.01 (0.94–1.08)	0.836	NA	NA
**Community connectedness** *(How much of your social time do you spend with LGBTQ+ friends or community?)*				
Almost all of the time	Ref	Ref	Ref	Ref

Most of the time	1.19 (0.98–1.45)	0.074	1.17 (1.01–1.36)	0.043
Some of the time	1.32 (1.09–1.59)	0.004	1.28 (1.10–1.49)	0.001
A little of the time	1.15 (0.93–1.43)	0.203	1.14 (0.97–1.35)	0.114
None of the time	NA	NA	NA	NA
**PrEP use**				
Daily PrEP	Ref	Ref	NA	NA
On demand PrEP	1.02 (0.94–1.09)	0.655	NA	NA
Lapsed**	0.89 (0.69–1.15)	0.370	NA	NA
Never taken PrEP	1.04 (0.97–1.12)	0.278	NA	NA
**Number of sexual partners in last 12 months**				
0–1	Ref	Ref	NA	NA
2–5	1.05 (0.92–1.20)	0.501	NA	NA
6–10	1.05 (0.92–1.20)	0.437	NA	NA
>10	1.00 (0.88–1.14)	0.968	NA	NA

HIV, human immunodeficiency virus; LGBTQ+, lesbian, gay, bisexual, transgender and queer people; NA, not applicable; PrEP, pre-exposure prophylaxis; Ref, reference level

* Australia's Medicare is a publicly funded universal healthcare system that provides free or subsidised access to medical services, hospital care, and prescription medications for Australian citizens and permanent residents

** Participants self-identifying as having previously taken PrEP but not currently using it were categorised as "lapsed".

Table S9: Comparison of test promoter characteristics between Wave 1 and Wave 2 (N=99)

	Wave 1 (n=91)	Wave 2 (n=8)	Total (n=99)	p value[†]
**Age (years)**				0.053
18–24	7 (7.7%)	2 (25.0%)	9 (9.1%)	
25–34	38 (41.8%)	5 (62.5%)	43 (43.4%)	
≥35	46 (50.5%)	1 (12.5%)	47 (47.5%)	
**Education level attained**				0.408
High school	3 (3.3%)	0 (0.0%)	3 (3.0%)	
Certificate/Diploma	12 (13.2%)	0 (0.0%)	12 (12.1%)	
Bachelor	38 (41.8%)	6 (75.0%)	44 (44.4%)	
Postgraduate	38 (41.8%)	2 (25.0%)	40 (40.4%)	
**First language other than English**				1.000
No	26 (28.6%)	2 (25.0%)	28 (28.3%)	
Yes	65 (71.4%)	6 (75.0%)	71 (71.7%)	
**Medicare status**				0.002
Medicare-eligible	64 (70.3%)	1 (12.5%)	65 (65.7%)	
Medicare-ineligible	27 (29.7%)	7 (87.5%)	34 (34.3%)	
**PrEP use**				0.378
Daily	32 (35.2%)	1 (12.5%)	33 (33.3%)	
On demand	39 (42.9%)	4 (50.0%)	43 (43.4%)	
Lapsed**	4 (4.4%)	0 (0.0%)	4 (4.0%)	
Never	16 (17.6%)	3 (37.5%)	19 (19.2%)	
**Community connectedness** *(How much of your social time do you spend with LGBTQ+ friends or community?)*				0.183[§]
Almost all of the time	5 (5.5%)	0 (0.0%)	5 (5.1%)	
Most of the time	36 (39.6%)	1 (12.5%)	37 (37.4%)	
Some of the time	39 (42.9%)	6 (75.0%)	45 (45.5%)	
A little of the time	11 (12.1%)	1 (12.5%)	12 (12.1%)	
None of the time	5 (5.5%)	0 (0.0%)	5 (5.1%)	
**HIV testing recency**				0.001[§]
<3 months ago	51 (56.0%)	1 (12.5%)	52 (52.5%)	
3–6 months ago	16 (17.6%)	0 (0.0%)	16 (16.2%)	
7–12 months ago	15 (16.5%)	2 (25.0%)	17 (17.2%)	
Over 12 months ago	9 (9.9%)	5 (62.5%)	14 (14.1%)	
Never tested for HIV	0 (0%)	0 (0%)	0 (0%)	
**Number of sexual partners in last 12 months**				0.544

0–1	5 (5.5%)	1 (12.5%)	6 (6.1%)	
2–5	26 (28.6%)	5 (62.5%)	31 (31.3%)	
6–10	19 (20.9%)	1 (12.5%)	20 (20.2%)	
>10	41 (45.1%)	1 (12.5%)	42 (42.4%)	
**Ease of instructions**				0.984§
Not easy	2 (2.2%)	1 (12.5%)	3 (3.0%)	
Moderately easy	11 (12.1%)	0 (0.0%)	11 (11.1%)	
Very easy	78 (85.7%)	7 (87.5%)	85 (85.9%)	
**Intention to use an HIV self-test kit again**				0.486§
Not likely	17 (18.7%)	1 (12.5%)	18 (18.2%)	
Moderately likely	29 (31.9%)	2 (25.0%)	31 (31.3%)	
Very likely	45 (49.5%)	5 (62.5%)	50 (50.5%)	

HIV, human immunodeficiency virus; LGBTQ+, lesbian, gay, bisexual, transgender and queer people; NA, not applicable; PrEP, pre-exposure prophylaxis; Ref, reference level

* Australia's Medicare is a publicly funded universal healthcare system that provides free or subsidised access to medical services, hospital care, and prescription medications for Australian citizens and permanent residents.

** Participants self-identifying as having previously taken PrEP but not currently using it were categorised as "lapsed".

† Fisher's exact test unless otherwise specified.

§ p values from ordered logistic regression.

Table S10: Comparison of recipient characteristics between Wave 1 and Wave 2 (N=260)

	Wave 1 (n=245)	Wave 2 (n=15)	Total (n=260)	p value[†]
**Age (years)**				<0.001
18–24	35 (14.3 %)	11 (73.3 %)	46 (17.7 %)	
25–34	129 (52.7 %)	2 (13.3 %)	131 (50.4 %)	
≥35	81 (33.1 %)	2 (13.3 %)	83 (31.9 %)	
**Education level attained**				<0.001
High school	14 (5.7%)	9 (60.0%)	23 (8.8%)	
Certificate/Diploma	24 (9.8%)	0 (0.0%)	24 (9.2%)	
Bachelor	129 (52.7%)	6 (40.0%)	135 (51.9%)	
Postgraduate	78 (31.8%)	0 (0.0%)	78 (30.0%)	
**First language other than English**				0.047
No	57 (23.3%)	0 (0.0%)	57 (21.9%)	
Yes	188 (76.7%)	15 (100.0%)	203 (78.1%)	
**Medicare status**				<0.001
Medicare-eligible	139 (56.7%)	0 (0.0%)	139 (53.5%)	
Medicare-ineligible	106 (43.3%)	15 (100.0%)	121 (46.5%)	
**PrEP use**				<0.001
Daily	56 (22.9%)	0 (0.0%)	56 (21.5%)	
On demand	95 (38.8%)	0 (0.0%)	95 (36.5%)	
Lapsed**	29 (11.8%)	0 (0.0%)	29 (11.2%)	
Never	65 (26.5%)	15 (100.0%)	80 (30.8%)	
**Community connectedness** *(How much of your social time do you spend with LGBTQ+ friends or community?)*				0.934[§]
Almost all of the time	34 (13.9 %)	0 (0 %)	34 (13.1 %)	
Most of the time	66 (27.0 %)	1 (6.7 %)	67 (25.8 %)	
Some of the time	95 (38.8 %)	6 (40.0 %)	101 (38.8 %)	
A little of the time	47 (19.2 %)	5 (33.3 %)	52 (20.0 %)	
None of the time	3 (1.2 %)	3 (20.0 %)	6 (2.3 %)	
**HIV testing recency**				<0.001[§]
<3 months ago	62 (25.3 %)	0 (0 %)	62 (23.8 %)	
3–6 months ago	71 (29.0 %)	0 (0 %)	71 (27.3 %)	
7–12 months ago	61 (24.9 %)	0 (0 %)	61 (23.5 %)	
Over 12 months ago	48 (19.6 %)	11 (73.3 %)	59 (22.7 %)	
Never tested for HIV	3 (1.2 %)	4 (26.7 %)	7 (2.7 %)	
**Number of sexual partners in last 12 months**				0.353[§]

0–1	39 (15.9%)	7 (46.7%)	46 (17.7%)	
2–5	80 (32.7%)	5 (33.3%)	85 (32.7%)	
6–10	57 (23.3%)	3 (20.0%)	60 (23.1%)	
>10	69 (28.2%)	0 (0.0%)	69 (26.5%)	
**Ease of instructions**				0.419§
Not easy	1 (0.4%)	0 (0.0%)	1 (0.4%)	
Moderately easy	34 (13.9%)	1 (6.7%)	35 (13.5%)	
Very easy	210 (85.7%)	14 (93.3%)	224 (86.2%)	
**Intention to use an HIV self-test kit again**				0.361§
Not likely	24 (9.8%)	1 (6.7%)	25 (9.6%)	
Moderately likely	54 (22.0%)	2 (13.3%)	56 (21.5%)	
Very likely	167 (68.2%)	12 (80.0%)	179 (68.8%)	

HIV, human immunodeficiency virus; LGBTQ+, lesbian, gay, bisexual, transgender and queer people; NA, not applicable; PrEP, pre-exposure prophylaxis; Ref, reference level

* Australia's Medicare is a publicly funded universal healthcare system that provides free or subsidised access to medical services, hospital care, and prescription medications for Australian citizens and permanent residents.

** Participants self-identifying as having previously taken PrEP but not currently using it were categorised as "lapsed".

† Fisher's exact test unless otherwise specified.

§ p values from ordered logistic regression.

Table S11: CONSORT checklist

Section/Topic	Item No	Checklist item	Reported on page No
**Title and abstract**			
	1a	Identification as a pilot or feasibility randomised trial in the title	1
	1b	Structured summary of pilot trial design, methods, results, and conclusions (for specific guidance see CONSORT abstract extension for pilot trials)	1
**Introduction**			
Background and objectives	2a	Scientific background and explanation of rationale for future definitive trial, and reasons for randomised pilot trial	3
	2b	Specific objectives or research questions for pilot trial	3
**Methods**			
Trial design	3a	Description of pilot trial design (such as parallel, factorial) including allocation ratio	4
	3b	Important changes to methods after pilot trial commencement (such as eligibility criteria), with reasons	4
Participants	4a	Eligibility criteria for participants	4
	4b	Settings and locations where the data were collected	4
	4c	How participants were identified and consented	4
Interventions	5	The interventions for each group with sufficient details to allow replication, including how and when they were actually administered	4
Outcomes	6a	Completely defined prespecified assessments or measurements to address each pilot trial objective specified in 2b, including how and when they were assessed	4
	6b	Any changes to pilot trial assessments or measurements after the pilot trial commenced, with reasons	NA
	6c	If applicable, prespecified criteria used to judge whether, or how, to proceed with future definitive trial	NA
Sample size	7a	Rationale for numbers in the pilot trial	4
	7b	When applicable, explanation of any interim analyses and stopping guidelines	NA
Randomisation:			
Sequence generation	8a	Method used to generate the random allocation sequence	NA
	8b	Type of randomisation(s); details of any restriction (such as blocking and block size)	NA
Allocation concealment mechanism	9	Mechanism used to implement the random allocation sequence (such as sequentially numbered containers), describing any steps taken to conceal the sequence until interventions were assigned	NA
Implementation	10	Who generated the random allocation sequence, who enrolled participants, and who assigned participants to interventions	NA
Blinding	11a	If done, who was blinded after assignment to interventions (for example, participants, care providers, those assessing outcomes) and how	NA
	11b	If relevant, description of the similarity of interventions	NA
Statistical methods	12	Methods used to address each pilot trial objective whether qualitative or quantitative	5
**Results**			

Participant flow (a diagram is strongly recommended)	13a	For each group, the numbers of participants who were approached and/or assessed for eligibility, randomly assigned, received intended treatment, and were assessed for each objective	6
	13b	For each group, losses and exclusions after randomisation, together with reasons	6
Recruitment	14a	Dates defining the periods of recruitment and follow-up	4
	14b	Why the pilot trial ended or was stopped	4
Baseline data	15	A table showing baseline demographic and clinical characteristics for each group	8
Numbers analysed	16	For each objective, number of participants (denominator) included in each analysis. If relevant, these numbers should be by randomised group	6,7
Outcomes and estimation	17	For each objective, results including expressions of uncertainty (such as 95% confidence interval) for any estimates. If relevant, these results should be by randomised group	6,7
Ancillary analyses	18	Results of any other analyses performed that could be used to inform the future definitive trial	NA
Harms	19	All important harms or unintended effects in each group (for specific guidance see CONSORT for harms)	NA
	19a	If relevant, other important unintended consequences	NA
**Discussion**			
Limitations	20	Pilot trial limitations, addressing sources of potential bias and remaining uncertainty about feasibility	14
Generalisability	21	Generalisability (applicability) of pilot trial methods and findings to future definitive trial and other studies	14
Interpretation	22	Interpretation consistent with pilot trial objectives and findings, balancing potential benefits and harms, and considering other relevant evidence	13
	22a	Implications for progression from pilot to future definitive trial, including any proposed amendments	14
**Other information**			
Registration	23	Registration number for pilot trial and name of trial registry	4
Protocol	24	Where the pilot trial protocol can be accessed, if available	NA
Funding	25	Sources of funding and other support (such as supply of drugs), role of funders	15
	26	Ethical approval or approval by research review committee, confirmed with reference number	4,15

Table S12: TREND checklist

Paper Section/Topic	Item No.	Descriptor	Reported? ✓	Pg #
**TITLE and ABSTRACT**				
Title and Abstract	1	• Information on how units were allocated to interventions	√	1
		• Structured abstract recommended		
		• Information on target population or study sample	√	1
**INTRODUCTION**				
Background	2	• Scientific background and explanation of rationale	√	2
		• Theories used in designing behavioral interventions		NA
**METHODS**				
Participants	3	• Eligibility criteria for participants, including criteria at different levels in recruitment/sampling plan (e.g., cities, clinics, subjects)	√	4
		• Method of recruitment (e.g., referral, self-selection), including the sampling method if a systematic sampling plan was implemented	√	4
		• Recruitment setting	√	4
		• Settings and locations where the data were collected	√	4
Interventions	4	• Details of the interventions intended for each study condition and how and when they were actually administered, specifically including:	√	4
		o Content: what was given?	√	4
		o Delivery method: how was the content given?	√	4
		o Unit of delivery: how were subjects grouped during delivery?		NA
		o Deliverer: who delivered the intervention?		NA
		o Setting: where was the intervention delivered?		NA
		o Exposure quantity and duration: how many sessions or episodes or events were intended to be delivered? How long were they intended to last?		NA
		o Time span: how long was it intended to take to deliver the intervention to each unit?	√	4
		o Activities to increase compliance or adherence (e.g., incentives)	√	4
Objectives	5	• Specific objectives and hypotheses	√	3
Outcomes	6	• Clearly defined primary and secondary outcome measures	√	5
		• Methods used to collect data and any methods used to enhance the quality of measurements	√	5
		• Information on validated instruments such as psychometric and biometric properties		NA
Sample size	7	• How sample size was determined and, when applicable, explanation of any interim analyses and stopping rules	√	4
Assignment method	8	• Unit of assignment (the unit being assigned to study condition, e.g., individual, group, community)	√	5
		• Method used to assign units to study conditions, including details of any restriction (e.g., blocking, stratification, minimization)		NA
		• Inclusion of aspects employed to help minimize potential bias induced due to non-randomization (e.g., matching)		NA
Blinding (masking)	9	• Whether or not participants, those administering the interventions, and those assessing the outcomes were blinded to study condition assignment; if so, statement regarding how the blinding was accomplished and how it was assessed		NA
Unit of Analysis	10	• Description of the smallest unit that is being analysed to assess intervention effects (e.g., individual, group, or community)	√	5

		• If the unit of analysis differs from the unit of assignment, the analytical method used to account for this (e.g., adjusting the standard error estimates by the design effect or using multilevel analysis)		NA
Statistical methods	1 1	• Statistical methods used to compare study groups for primary methods outcome(s), including complex methods for correlated data	√	5
		• Statistical methods used for additional analyses, such as subgroup analyses and adjusted analysis	√	5
		• Methods for imputing missing data, if used		NA
		• Statistical software or programs used	√	5
**RESULTS**				
Participant flow	1 2	• Flow of participants through each stage of the study: enrollment, assignment, allocation and intervention exposure, follow-up, analysis (a diagram is strongly recommended)	√	6+Fig2
		o Enrollment: the numbers of participants screened for eligibility, found to be eligible or not eligible, declined to be enrolled, and enrolled in the study	√	6
		o Assignment: the numbers of participants assigned to a study condition	√	6
		o Allocation and intervention exposure: the number of participants assigned to each study condition and the number of participants who received each intervention		NA
		o Follow-up: the number of participants who completed the follow-up or did not complete the follow-up (i.e., lost to follow-up), by study condition	√	6
		o Analysis: the number of participants included in or excluded from the main analysis, by study condition	√	6
		• Description of protocol deviations from study as planned, along with reasons		NA
Recruitment	1 3	• Dates defining the periods of recruitment and follow-up		4
Baseline data	1 4	• Baseline demographic and clinical characteristics of participants in each study condition	√	Table 1
		• Baseline characteristics for each study condition relevant to specific disease prevention research		NA
		• Baseline comparisons of those lost to follow-up and those retained, overall and by study condition		NA
		• Comparison between study population at baseline and target population of interest		NA
Baseline equivalence	1 5	• Data on study group equivalence at baseline and statistical methods used to control for baseline differences	√	NA
Numbers analyzed	1 6	• Number of participants (denominator) included in each analysis for each study condition, particularly when the denominators change for different outcomes; statement of the results in absolute numbers when feasible	√	6
		• Indication of whether the analysis strategy was "intention to treat" or, if not, description of how non-compliers were treated in the analyses		NA
Outcomes and estimation	1 7	• For each primary and secondary outcome, a summary of results for each estimation study condition, and the estimated effect size and a confidence interval to indicate the precision	√	6-7
		• Inclusion of null and negative findings		NA
		• Inclusion of results from testing pre-specified causal pathways through which the intervention was intended to operate, if any		NA
Ancillary analyses	1 8	• Summary of other analyses performed, including subgroup or restricted analyses, indicating which are pre-specified or exploratory		NA
Adverse events	1 9	• Summary of all important adverse events or unintended effects in each study condition (including summary measures, effect size estimates, and confidence intervals)		NA
**DISCUSSION**				
Interpretation	2 0	• Interpretation of the results, taking into account study hypotheses, sources of potential bias, imprecision of measures, multiplicative analyses, and other limitations or weaknesses of the study	√	11
		• Discussion of results taking into account the mechanism by which the intervention	√	11

		was intended to work (causal pathways) or alternative mechanisms or explanations		
		• Discussion of the success of and barriers to implementing the intervention, fidelity of implementation		NA
		• Discussion of research, programmatic, or policy implications	√	12
Generalizability	21	• Generalizability (external validity) of the trial findings, taking into account the study population, the characteristics of the intervention, length of follow-up, incentives, compliance rates, specific sites/settings involved in the study, and other contextual issues	√	12
Overall evidence	22	• General interpretation of the results in the context of current evidence and current theory	√	12
